# Electrochemical detection and removal of brilliant blue dye *via* photocatalytic degradation and adsorption using phyto-synthesized nanoparticles†

**DOI:** 10.1039/d3ra07519b

**Published:** 2024-01-12

**Authors:** Kashif Ali Khan, Afzal Shah, Jan Nisar

**Affiliations:** a Department of Chemistry Quaid-i-Azam University Islamabad 45320 Pakistan afzals_qau@yahoo.com; b National Centre of Excellence in Physical Chemistry, University of Peshawar Peshawar 25120 Pakistan

## Abstract

Herein, we report a sensitive electrochemical platform prepared by modifying the electrode surface with copper-doped zinc oxide nanoparticles; these nanoparticles were prepared *via* a green synthetic approach using the extract of *Cassia fistula* leaves and multiwalled carbon nanotubes (MWCNTs). For the best response of the electrode modifier, a number of experimental conditions were optimized to obtain the most intense signal of the target analyte Coomassie brilliant blue using a rapid analysis technique square wave voltammetry. The designed sensor displayed remarkable sensitivity for Coomassie brilliant blue with a detection limit of 0.1 nM under the optimized conditions. Moreover, the repeatability, specificity and reproducibility of the designed sensor demonstrated its potential for practical applications. The sensing platform was also used for monitoring the degradation kinetics of the Coomassie brilliant blue dye. Catalytic degradation of the dye was performed using the synergistic effect of Cu–ZnO NPs together with Fenton reagent. The dye degraded by 96% in 60 minutes under neutral conditions, which is one of the main achievements of this work that has never been reported. The photocatalytic breakdown of Coomassie brilliant blue was also monitored using UV-visible spectroscopy. The degradation kinetics results of both techniques agreed well. The adsorption of Coomassie brilliant blue using ZnO NPs was monitored spectrophotometrically. The adsorption data were fitted in a pseudo-second order kinetic model by following the Langmuir isotherm at lower concentration and Freundlich isotherm at higher concentration.

## Abstract 2

A sensitive electrochemical platform is sensing brilliant blue dye and monitors its removal from wastewater by degradation and adsorption processes.

## Graphical abstract

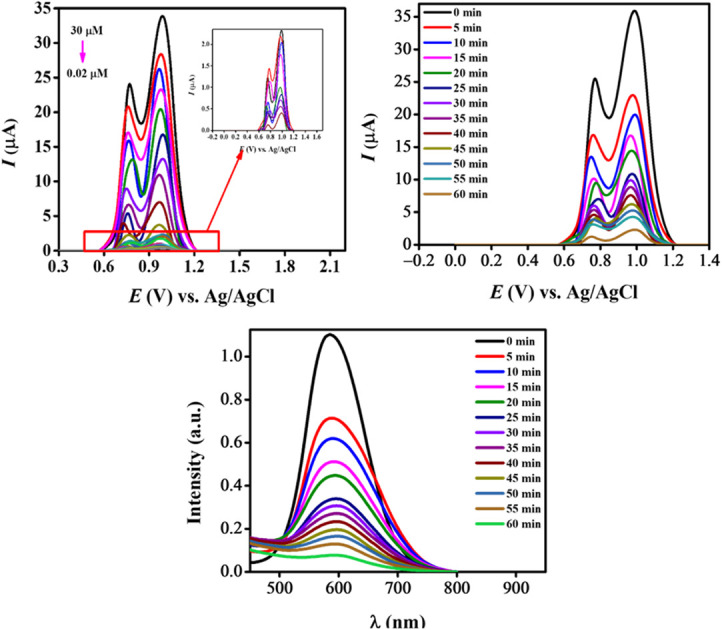

## 1. Introduction

Water is essential for all living organisms on earth. The presence of water contaminants is a serious threat to organisms living in water and on land. The escalating industrialization over the past century has embellished human life with luxuries; however, it also led to environmental pollution. The untreated discharge of contaminated industrial effluents has badly affected the ecosystem. Among a variety of environmental contaminants, dyes are a major source of pollution that jeopardize aquatic and terrestrial biota. Therefore, researchers have been seriously engaged in developing effective, affordable and eco-friendly methods to determine and eliminate hazardous dyes. The unregulated industrial discharge of effluents is an environmental issue of great concern because wastewaters of textile, paint and pigment industries are loaded with toxic dyes. Dyes exert adverse effects on both water sources and aquatic organisms. More than 10 000 industrial dyes are utilized in different industrial sectors such as paper, tanning, textile, cosmetics, plastic, and pharmaceuticals.^1^ Despite an annual production rate exceeding 700 000 tons, it is estimated that 10% of dyes are wasted in industrial activities, which are believed to have a harmful impact on natural water supplies. Many environmental concerns are brought about by the untreated discharge of contaminated effluent into the ecosystem, including reduced photosynthesis and the extinction of aquatic biota.^2^

Coomassie brilliant blue R-250 (CBB R-250), also called as brilliant blue dye,^3^ is an anionic synthetic dye commonly termed as disulphonated triphenylmethane dye owing to the presence of three phenyl rings in its structure. CBB R-250 was initially developed for use in the textile industry but is now majorly used as a basis of stain for the detection of protein in gel electrophoresis in the field of analytical biochemistry.^4^ Besides a number of useful applications, it poses some severe threats to human beings and other living organisms. It belongs to a class of organic compounds termed as triphenylmethane dyes that have been widely reported for mutagenic and teratogenic effects on living organisms.^5^ The Material Safety Data Sheet (MSDS) lists CBB R-250 as a potential irritant to the skin, eyes, and respiratory system. Prolonged or repeated exposure can lead to skin sensitization, and inhaling the dye may cause respiratory irritation. According to MSDS, the dye possesses potential damage if ingested, leading to gastrointestinal discomfort, nausea, vomiting, and diarrhea.^6^ In light of the literature review, CBB R-250 is a novel dye in the field of sensing, and no work has been reported so far for its detection despite having these potential toxic effects. The presence of an electron-rich moiety and conjugated system in the structure of CBB R-250 makes it a suitable analyte for detection through UV-visible spectroscopy and voltammetric techniques. The current work presents the detection and removal of CBB R-250 dye from contaminated water sources using a highly sensitive electrochemical sensor composed of MWCNTs and Cu–ZnO modified glassy carbon electrode (GCE). The reasons for the choice of these materials are given in the subsequent paragraphs.

MWCNTs have emerged as a promising choice to be used as an electrochemical sensor because of their exceptional electrical conductivity, unique structural properties and high surface area. The use of MWCNTs as an electrochemical sensor has been widely reported for different analytes. Moreover, previous studies have demonstrated the enhanced sensitivity of the modified electrode resulting from the combined use of MWCNTs and NPs.^7^ This phenomenon can be ascribed to the substantial increase in surface area facilitated by the NPs. ZnO is an important semiconductor material that generally has an exciton binding energy of roughly 60 meV and possesses a 3.37 eV band gap energy in normal conditions.^8^ This material holds significant importance in the field of semiconductors and is extensively utilized in many applications such as optical devices, piezoelectric devices, photocatalysis, chemical sensors, and transparent conductors.^9^ Several studies have been undertaken for the modification of characteristics of ZnO NPs to render them suitable for diverse uses. The modification of specific features, such as structural, optical, magnetic and electrical properties in ZnO can be achieved through the selective doping of various elements, particularly transition metal ions like iron, copper, cobalt, and others. The introduction of Cu^2+^ into the ZnO lattice brings forth a wide range of appealing characteristics, such as improved electrical conductivity, modified optical properties, and in certain instances, the manifestation of ferromagnetism.^10^ The aforementioned modifications present novel opportunities to utilize Cu–ZnO NPs in a diverse range of fields. These qualities offer notable benefits, especially in the context of electrochemical sensing applications.^11^ This research paper focuses on the fascinating realm of the synergistic effects achieved by combining MWCNTs and Cu–ZnO NPs where the distinct advantages of both materials are harnessed to create an electrochemical sensor that holds great promise for the selective and sensitive detection of CBB R-250.

The process of urbanization and industrial growth in developing nations has been found to have negative consequences on both the environment and human well-being. Every year, a substantial amount of chemicals, amounting to billions of tons, are released into the environment by various industries, such as oil, gas, and textile.^12^ The presence of dyestuff in the effluents has been found to have detrimental effects on the environment and the long-term viability of life on Earth.^13^ Several methods (including ozonolysis, photocatalysis, membrane coagulation, chemical degradation and adsorption) have been employed for the elimination of these contaminants from water sources.^14^ The current work mainly focuses on photocatalytic degradation and adsorption methods owing to their environmental friendliness and economical behavior. K. Singh *et al.* employed green synthesized ZnO NPs for the photodegradation of CBB R-250, and achieved 93% degradation in 3 hours.^4^ Venkata *et al.* reported on 58.38% degradation of CBB R-250 in 90 minutes using CuO NPs.^15^ N. Kaur *et al.* evaluated the performance of NiO NPs for the photodegradation of CBB R-250 dye, which resulted in 95.7% degradation in 140 minutes. Magsino *et al.* used iron oxide-graphene oxide composite for the adsorptive study of CBB R-250, and reported an adsorption capacity of 14.31 mg g^−1^.^16^ Sharma *et al.* used starch/poly(alginic acid-*cl*-acrylamide) nanohydrogel, and evaluated an adsorption capacity of 31.24 mg g^−1^ towards the dye.^17^ S. Nayak *et al.* demonstrated 94.79% adsorption of the dye by using Au–Ag core shell nanoparticles.^18^ ZnO is a widely used photocatalyst owing to its tunable band gap. Copper doping to the ZnO lattice can significantly reduce the band gap, as well as minimize the rate of charge recombination. We used green synthesized Cu–ZnO NPs for the photodegradation of CBB R-250 dye in synergy with the Fenton reagent that demonstrated 96% degradation in 60 min under neutral conditions. The performance of pristine ZnO NPs was also tested towards adsorption of the dye, which demonstrated an adsorption capacity of 48 mg g^−1^.

This study reports the voltammetric detection of CBB R-250 dye by using MWCNTs/Cu–ZnO/GCE for the first time. The modified electrode was also used for monitoring the photocatalytic degradation of the dye. To date, no study has been reported so far for the detection of this particular dye. A drop casting method was used to immobilize the analyte over the surface of the modified electrode. This method allows for an intense peak of the analyte by minimizing the diffusion and orientation barriers. Also, this method has proven to be environment friendly, as only a small amount of analyte is used. In contrast, the conventional method involves the analyte being taken in bulk in solution, which can cause toxic effects if disposed without pretreatment. Adsorption of the dye was also monitored using UV-vis spectrophotometric techniques. Also, the literature is devoid of any document for the nanoparticles-assisted photo-Fenton degradation of CBB R-250 under neutral conditions. Furthermore, our study presents adsorption of the dye using a novel green synthesized adsorbent. The present article is the first effort to achieve and present the three objectives of CBB R-250 dye detection, degradation, and adsorption in a single document.

## 2. Experimental

### 2.1. Materials

All of the materials used throughout this work were of analytical grade. A list of chemicals along with their percent purity and suppliers can be seen in Table S1.†

### 2.2. Instrumentation

The surface morphology and elemental analysis was carried out using a JOEL-JSM-IT100 device with an operational voltage of 20 kV. The optical properties of pristine and Cu–ZnO NPs were investigated utilizing a UV-vis spectrophotometer (Shimadzu 1700). PL spectra of the synthesized NPs were obtained utilizing the PerkinElmer LS 55 Luminescence spectrometer. Structural information (crystalline structure) was obtained using the PANalytical X-ray diffractometer model 3040/60 X'Pert PRO operated at 45 kV and 40 mA. The FTIR spectra were recorded using a Shimadzu 8400S FTIR spectrometer having a range of 4000 to 400 cm^−1^.

### 2.3. Synthesis of ZnO and Cu–ZnO NPs

In this work, ZnO NPs and doped ZnO NPs were phytosynthesized using an environment-friendly green synthesis route utilizing the leaf extract of *Cassia fistula*. Initially, the leaves of the said plant were carefully collected and subjected to a thorough washing process using distilled water to remove any sort of impurity. Subsequently, the leaves were spread out and allowed to air dry for approximately one week. Once it was confirmed that all of the leaves had completely dried, they were finely powdered through grinding. Powdered leaves were then mixed with distilled water in a ratio of 1 : 100 and heated at 90–100 °C for about 4 hours. The resulting extract was filtered and centrifuged to eliminate any insoluble materials. The purified extract was stored at 4 °C for future use.

**Scheme 1:**
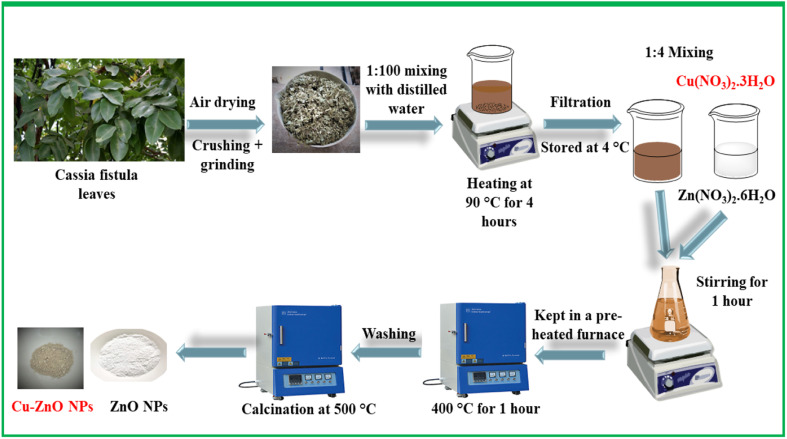
Eco-friendly approach for the phytosynthesis of undoped ZnO NPs and Cu–ZnO NPs.

The synthesis of ZnO NPs involved a reaction between a mixture of 0.1 M Zn(NO_3_)_2_·6H_2_O and the plant extract in a ratio of 4 : 1. The reaction mixture was stirred for an hour, followed by heat treatment at 400 °C for 1 hour in a pre-heated furnace. During the reaction, the metal nitrate part obtained from Zn(NO_3_)_2_·6H_2_O acted as an oxidizing agent, while the organic part obtained from *Cassia fistula* leaves extract acted as a reducing agent. The synthesized ZnO NPs were then obtained, and washed thoroughly with ethanol and distilled water to remove any sort of impurities. Finally, ZnO NPs were calcined at 500 °C for 2 hours and stored in an airtight bottle for further use. The same synthetic scheme (Scheme 1) was followed for the synthesis of Cu–ZnO, while a molar ratio of Cu(NO_3_)_2_·3H_2_O was introduced along with Zn(NO_3_)_2_·6H_2_O during the first step. The rest of the procedure was the same as that described above.

### 2.4. Electrode modification

Prior to the modification of the electrode, it was physiochemically cleaned to achieve a shiny surface for GCE. It was achieved by rubbing it over an alumina slurry using a nylon rubber mat in a manner of digit eight to remove any scratches or impurities that might be present over the surface of the electrode. The physiochemically cleaned GCE was then sonicated in a mixture of ethanol, water, and acetone to get rid of the contamination from the surface of the electrode that might have been attached during the process of polishing. Then, it was dried under ambient conditions. Then, the electrode was cleaned electrochemically by subjecting the bare electrode to cyclic voltammograms in a range of 0.1–0.5 V until reproducible results were produced. This was done to remove any sort of oxidizable impurity present over the surface of the electrode.

For the modification of GCE, 1 mg/1 mL slurries of Cu–ZnO NPs and MWCNTs were prepared in dimethylformamide (DMF) and ultra-sonicated for about 3 hours. The electrode was then fabricated with layer-by-layer deposition of both modifiers using a 10 μL drop of each, followed by a 10 μL drop of the analyte. For each of the depositions, the electrode was air-dried. Upon complete drying of the electrode after fabrication with the analyte, it was placed in the electrochemical cell and voltammetric analysis was performed. The cell assembly utilized for the electrochemical detection and degradation study primarily consisted of an electrochemical cell and a three-electrode system (reference, counter and working electrode). The electrochemical cell of 20 mL capacity was made up of double-walled glass mounted with Teflon cell top. The electrochemical measurements were performed using a Multi-Channel Metrohm Autolab (Galvanostat/Potentiostat) (Utrecht, The Netherlands) operated with NOVA 1.11 software.

### 2.5. Dye degradation method

The photodegradation of the CBB R-250 was achieved using a NPs assisted photo-Fenton reaction. For that purpose, 2 mg dose of Cu–ZnO NPs were dispersed to 25 mL of the dye solution. Prior to the addition of NPs, the concentration of the dye was optimized at 30 μM to have the UV-vis absorbance near 1. After the addition of NPs, 2–3 mg iron salt having iron in the +2 oxidation state (FeCl_2_) was also added to the reaction mixture, along with the addition of 2 mL H_2_O_2_. Then, CBB R-250 solution was stirred for about 10 minutes in the dark to make it homogenous, and subsequently placed in direct sunlight (in the months of May–June, 12 PM onwards) to monitor the degradation process. The degradation of the dye was monitored both electrochemically and spectrophotometrically. For spectroscopic analysis, a 2 mL sample was obtained from the reaction mixture at regular time intervals, while a 50 μL sample was obtained and stored under dark conditions so as to stop the photocatalytic process. A 10 μL drop from these samples was then immobilized on the modified GCE for the voltammetric analysis.

### 2.6. Dye adsorption method

Adsorption of the dye was spectrophotometrically monitored using UV-vis spectroscopy. For that purpose, a 10 mg dose of the ZnO NPs was added to the 25 mL dye solution, while the reaction mixture was completely covered with the help of aluminium foil so as to ensure complete darkness for the adsorption process to occur. Constant stirring was maintained, and the UV-vis spectra were obtained after constant time intervals. pH optimization was performed using 0.1 M solution of NaOH and HCl.

## 3. Results and discussion

### 3.1. Characterization of undoped and Cu-doped ZnO NPs

The structural, optical, and morphological properties of the synthesized pristine ZnO and Cu–ZnO NPs were studied using UV-vis, PL, FTIR, XRD, SEM, and EDS analysis.

**Fig. 1:**
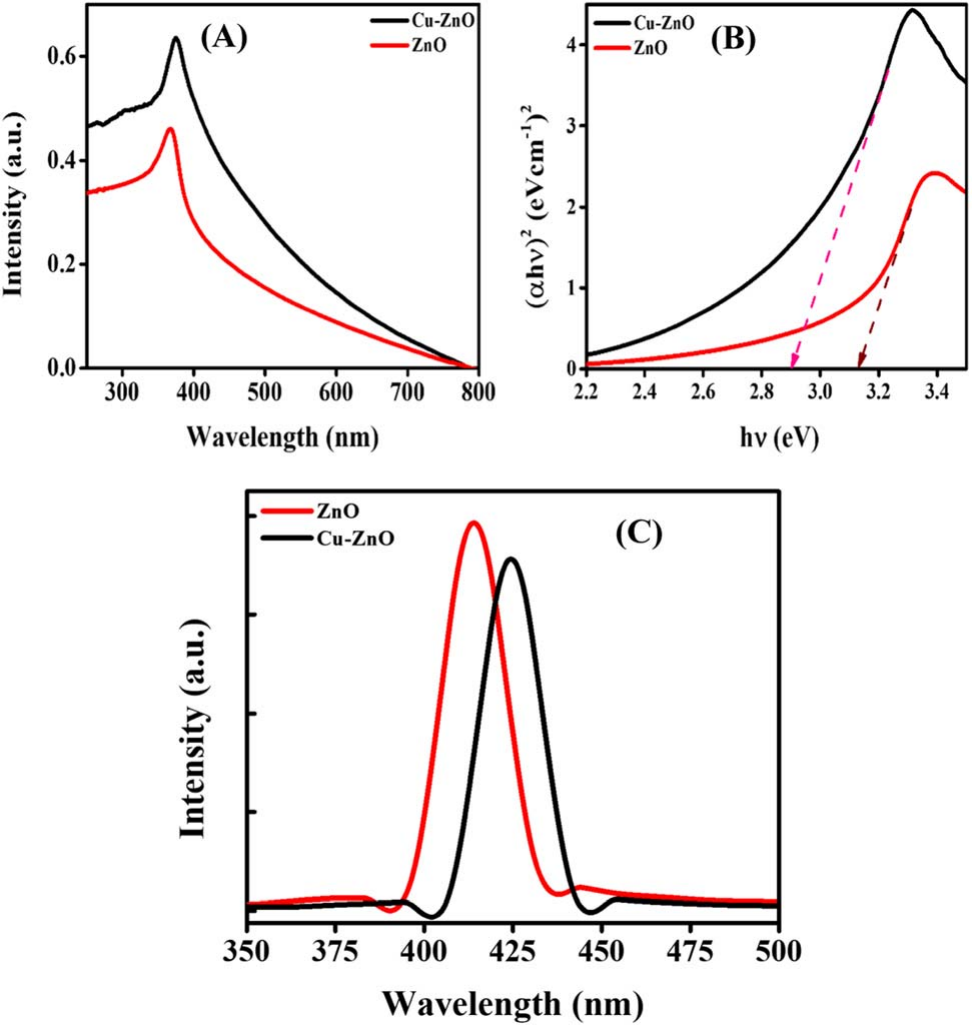
(A) UV-visible absorption spectra; (B) Tauc plots; and (C) PL emission spectra of ZnO and Cu–ZnO NPs.

The characteristic peak of ZnO NPs was noticed at 367 nm, which was in accordance with the literature.^19^ There was a significant red-shift in the case of Cu–ZnO NPs that gave the absorption-maxima (*λ*_max_) at 375 nm, as shown in Fig. 1A. This increase in the *λ*_max_ towards higher wavelength was observed because of the generation of some extra energy levels due to the inclusion of Cu ions into the lattice of ZnO. The Tauc plot approach was employed to determine the band gap, as illustrated in Fig. 1B. The following formula was employed to estimate the band gap value.^20^

1 (*αhυ*)^*n*^ = *A*(*hυ* − *E*_g_)

where, “*A*” denotes the proportionality constant, the symbol “*h*” is the Planck's constant, “*ν*” symbolizes the frequency of photons, “*α*” signifies the absorption coefficient, and “*E*_g_” refers to the band gap energy. The value of the parameter “*n*” for direct transition band gaps is 2, while the value for an indirect band gap is 1/2. The band gap of ZnO NPs was determined to be 3.17 eV. However, upon doping with Cu, the band gap decreased to 2.9 eV.

PL spectra of the phytosynthesized ZnO and Cu–ZnO NPs are shown in Fig. 1C. A laser beam was used to excite the electrons from their ground state to their excited state. The light being emitted from those excited electrons was then captured as they fell back to the ground state. It was observed that ZnO gave an emission maximum at 414 nm, while there was a red shift being recorded for the Cu–ZnO NPs having *λ*_max_ for emission at 424 nm. This violet emission could possibly be because of the transition from the conduction band edge to zinc vacancy.^21^ While in the case of Cu–ZnO NPs, there is production of a Cu-defect below the ZnO conduction band edge in the energy levels of ZnO, which is responsible for the shift of the emission spectra towards higher wavelength.^22^ The intensity of the peak was decreased for Cu–ZnO NPs, which specifies a low recombination rate of the charge carriers in ZnO NPs with increasing concentration of the dopant, and hence improved photocatalytic properties.

**Fig. 2:**
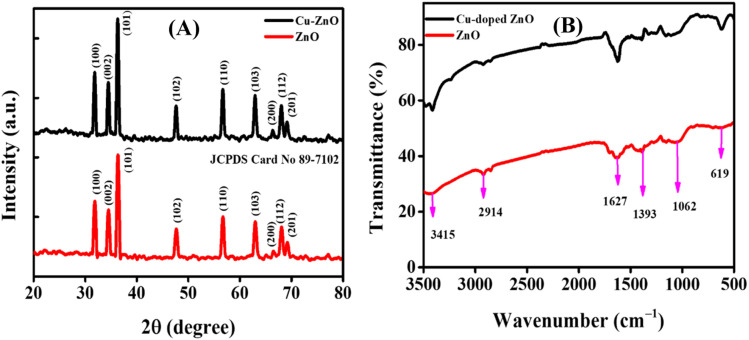
(A) XRD pattern; and (B) FTIR spectra of ZnO and Cu–ZnO NPs.

The XRD patterns were analyzed for structural features and phase purity. Fig. 2A presents the XRD spectra of both ZnO and Cu–ZnO NPs. The graph shows distinct diffraction peaks, which provides evidence that the synthesized NPs possess a crystalline structure. These peaks can be attributed to specific crystallographic planes, namely (100), (002), (101), (102), (110), (103), (200), (112), and (201), as determined by comparison with the JCPDS card no. 89-7102. Additionally, both samples exhibit a hexagonal wurtzite phase, with a preferred orientation along the (101) plane.^23^ In the case of Cu–ZnO NPs, a minor displacement of peaks towards lower theta values was found. Additionally, no new phase of dopant was detected, indicating the occurrence of substitutional doping in the current scenario. The aforementioned observation suggests that the integration of Cu^2+^ ions into the ZnO lattice is achieved with a high level of effectiveness, without inducing any disturbances to the overall crystal structure. The average sizes of the crystallites in the nanostructures was calculated by employing the Debye–Scherrer formula, which is expressed as,^24^2
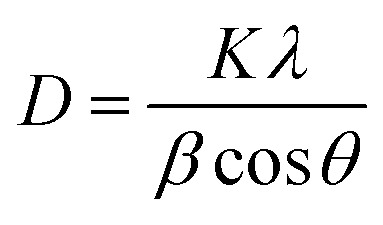
where *D*, *θ*, *β*, *K*, and *λ* refer to the average size of the crystallites of particles, peak position (Braggs angle), peak full width-half maximum (FWHM) value in radian, Scherrer constant that has a numerical value of 0.94, and the wavelength of X-rays (Cu Kα = 0.15406 nm) respectively. The average sizes of the crystallites for ZnO NPs and Cu–ZnO NPs were calculated to be 18.94 nm and 21.70 nm, respectively.

FTIR spectroscopy was used to examine the functional groups of the produced NPs. Various phytochemicals, such as tannins, saponins, triterpenoids, steroids, anthraquinones, glycosides, flavonoids, reducing sugars, proteins, amino acids, and carbohydrates, present in the leaf extract of *Cassia fistula* have been determined using biochemical and HPLC techniques.^25^ These phytochemicals function as stabilizing/capping and reducing agents during the phytofabrication of NPs. Fig. 2B depicts the FTIR spectra of the fabricated ZnO NPs and Cu–ZnO NPs. The spectral peak at 619 cm^−1^ corresponds to the Zn–O stretching vibration, which proposed the formation of ZnO NPs. The peak at 1627 cm^−1^ corresponds to the C=O stretching vibration of the amide carbonyl group, and points towards the presence of proteins. The sharp peak at 1062 cm^−1^ is due to the C–O stretching vibration present in the carbohydrates, while the peak at 3415 cm^−1^ corresponds to the O–H stretching vibration. The peak at 1393 cm^−1^ originated due to the sp^3^ C–H bending vibration, and was well complemented by a peak at 2914 cm^−1^ that corresponds to the sp^3^ C–H stretching vibration.^22^

**Fig. 3:**
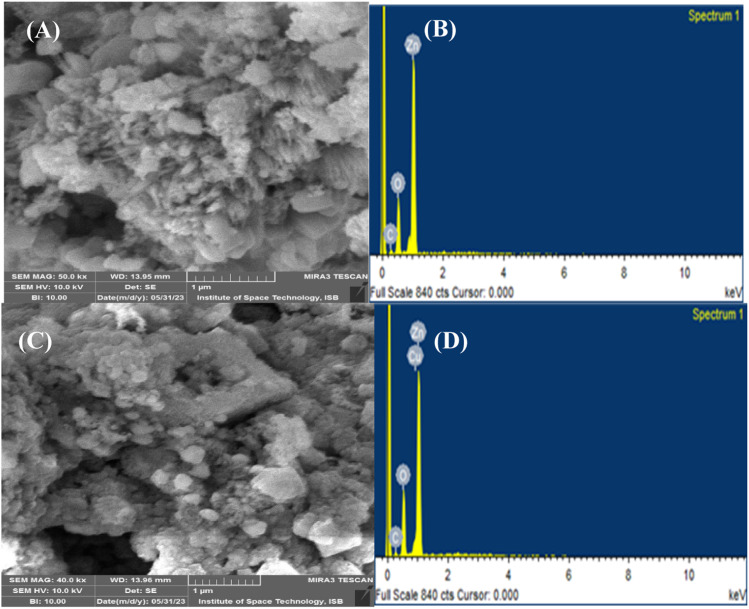
SEM micrographs of (A) pure ZnO NPs; (C) Cu–ZnO NPs; EDS spectra of (B) pure ZnO NPs; (D) Cu–ZnO NPs.

The morphological characteristics of the synthesized NPs were assessed by carrying out SEM analysis, as depicted in Fig. 3. The SEM micrographs of pristine ZnO NPs (Fig. 3A) and Cu–ZnO NPs (Fig. 3C) revealed no specific morphology, while the particles are irregularly scattered having agglomeration. Some of the spherical and fibrous particles are also present, but these are quite low in number. Therefore, it is quite difficult to calculate the particle size using these SEM images. The elemental makeup and purity of pure and Cu–ZnO NPs were determined by employing EDS analysis, the results of which are shown in Fig. 3B and D. The elements like Zn, O, and Cu appeared as the major constituents, while the minor peak of C in both cases can be linked to the carbon ribbon that is used during EDS study. A comprehensive elemental formulation of the phytosynthesized nanostructure is given in Table S2.†

### 3.2. Electrochemical characterization

Voltammetric techniques such as CV and EIS serve as useful tools for the electrochemical characterization of the fabricated electrodes. The electrochemical techniques and their findings are discussed in the forthcoming sections.

**Fig. 4:**
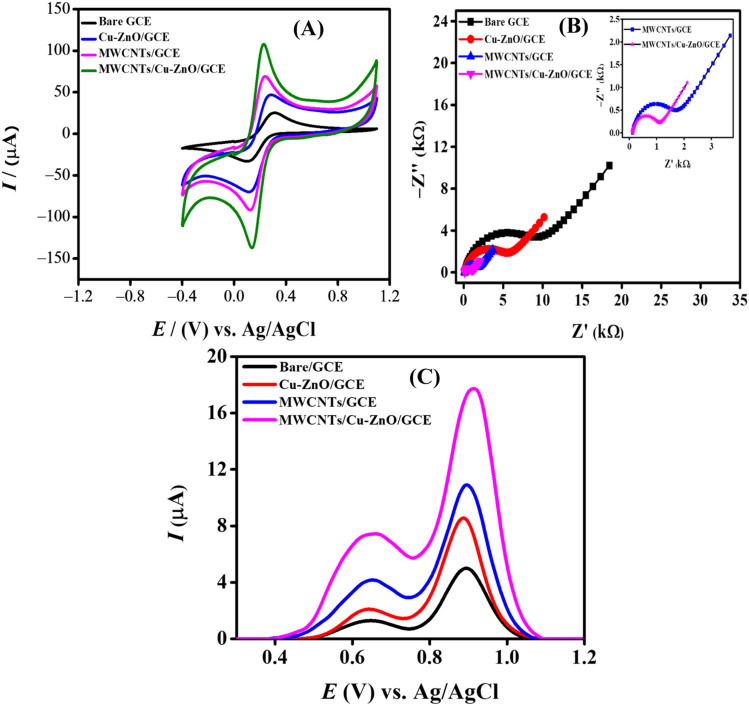
(A) Cyclic voltammograms at bare and modified GCEs; (B) Nyquist plots obtained from EIS data; (C) SWVs of modified GCEs in PBS (pH = 6).

A significant factor that governs the electrochemical sensing scaffold's performance is the area of the electrode, which can strongly influence the peak current response. For investigating the change in the electroactive surface area, cyclic voltammograms were obtained in the presence of a 0.005 M solution of the redox probe K_3_[Fe(CN)_6_] in 0.1 M KCl (supporting electrolyte) at 100 mV s^−1^ scan rate. CV was employed for the electrochemical characterization of the fabricated electrodes. The cyclic voltammetric responses of GCE, Cu–ZnO/GCE, MWCNTs/GCE, and MWCNTs/Cu–ZnO/GCE were investigated, as shown in Fig. 4A. There was greater current and better reversibility exhibited for all the modified electrodes compared to bare GCE, the best of them being MWCNTs/Cu–ZnO/GCE. The redox behavior of the K_3_[Fe(CN)_6_] was observed for the differently modified GCEs. The Randles–Sevcik equation (eqn (3)), was used to determine the electrode's electroactive surface area.^26^

3 *I*_pa_ = 2.6 × 10^5^*n*^3/2^*D*^1/2^*Cυ*^1/2^*A*

Here, *I*_pa_ denotes the anodic peak current in amperes, *D* denotes the diffusion coefficient in cm^2^ s^−1^, *n* shows the number of electrons, *ν* symbolizes the scan rate in V s^−1^, *A* symbolizes the electroactive surface area of GCE in cm^2^, and *C* represents the concentration of K_3_[Fe(CN)_6_]. For potassium ferricyanide, *D* = 7.6 × 10^−6^ cm^2^ s^−1^ and *n* = 1. The surface area determined for unmodified and modified GCEs is given in Table S3.† After modification, MWCNTs/Cu–ZnO/GCE demonstrated a fourfold enhancement in electroactive surface area in contrast to the unaltered GCE. MWCNTs/Cu–ZnO/GCE offers a greater number of binding sites for CBB R-250 molecules, consequently facilitating the expedited transfer of electrons. Additionally, the increased electrocatalytic activity of MWCNTs/Cu–ZnO/GCE in comparison to bare GCE is evidenced by the reduced peak separation value and maximum peak current response.

The interfacial properties of the electrode–electrolyte interface were studied using the EIS spectra of the various modified electrodes. An EIS spectrum is composed of two parts, *i.e.*, a semicircle at a higher frequency region termed as charge transfer resistance (*R*_ct_), while a linear portion that is 45° to the semicircle is called the Warburg resistance (*Z*_w_).^20^*R*_ct_ and *Z*_w_ are related to the electron transfer and mass transfer that occur at the interface of the electrode and electrolyte. Fig. 4B shows the model-fitted data of Nyquist plots obtained from the EIS spectra of various modified electrodes using 0.005 M K_3_[Fe(CN)_6_] as a redox probe in 0.1 M solution of KCl as a supporting electrolyte. The frequency was varied from 100 000 Hz to 0.1 Hz with a 10 mV signal amplitude. A larger semicircular portion having a *R*_ct_ value of 8820 Ω was observed for the bare GCE, corresponding to a high resistance to charge transfer between GCE and electrolyte. The semicircular part exhibited a sequential decrease for the modified electrodes with a consequent decrease in *R*_ct_ value with the smallest of them being 924.2 Ω for MWCNTs/Cu–ZnO/GCE, indicating a facile charge transfer being offered at the surface of this modified GCE. Randles' equivalent circuit was employed to fit the EIS results to calculate different parameters. The schematic diagram presented in Fig. S1† illustrates the utilization of an equivalent circuit model (RCRW) for the purpose of fitting the experimental data. This model incorporates the Warburg impedance, resistors, and a constant phase element. It was observed that the *R*_ct_ value decreased for MWCNTs/Cu–ZnO/GCE as compared to bare GCE and offered low impedance during the charge transfer, resulting in faster electron transfer during the electrochemical reactions. The EIS parameters obtained using the fitted data for GCE, Cu–ZnO/GCE, MWCNTs/GCE, and MWCNTs/Cu–ZnO/GCE are shown in Table S4.†

### 3.3. Voltammetric behavior of CBB R-250 dye

Square wave voltammograms were recorded in a phosphate buffer solution (PBS) of pH 6 using various modified GCEs, while using a 10 μL drop of 30 μM dye in each case. SWV were recorded keeping the electrochemical window from 0 V to 1.5 V; the deposition time and accumulation potential were 5 s and 0 V, respectively. It can be observed from Fig. 4C that MWCNTs/Cu–ZnO/GCE gave the maximum current response as compared to MWCNTs/GCE, Cu–ZnO/GCE, and bare GCE. As evident from the EIS and CV data, MWCNTs/Cu–ZnO/GCE offered less charge transfer resistance and an improved surface area, so the oxidation of CBB R-250 over the electrode surface was greatly facilitated by using our modified sensing platform. MWCNTs are known for their high surface area and excellent electrical conductivity, which promotes the oxidation of dye over electrode surface. The combined effect of Cu–ZnO NPs and MWCNTs is responsible for a high current response of the GCE towards the dye due to the presence of a highly porous structure at the surface of GCE. This facilitates the adsorption of dye over the surface, while Cu–ZnO NPs also act as a catalyst for the electro-oxidation of CBB R-250 by lowering the activation energy. MWCNTs are known for their extended π electron system, which interacts with the aromatic rings of CBB R-250 through π–π stacking interaction that further affects the adsorption of dye onto the MWCNTs surface, potentially influencing the electrochemical behavior of the system.^27^ So overall, the enhanced surface area, low charge transfer resistance, catalytic behavior of Cu–ZnO NPs, and interaction of MWCNTs with the dye result in the adsorption of CBB R-250 dye over MWCNTs/Cu–ZnO/GCE, which facilitates the oxidation of the dye.

The effects of the scan rate and optimization of various experimental conditions have been described in Section S1 and S2,† respectively.

### 3.4. Analytical applications of the developed sensor

#### 3.4.1. Assessment of the detection and quantification limits of CBB R-250

**Fig. 5:**
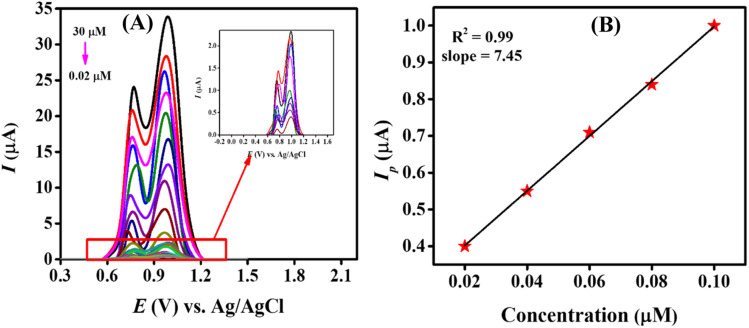
(A) SW voltammograms of different concentrations of CBB R-250 varying from 30 μM to 0.02 μM using MWCNTs/Cu–ZnO/GCE in 0.3 M H_2_SO_4_; (B) linearity plot obtained from the lower concentrations of the analyte.

The sensitivity and stability of our designed sensing scaffold were determined by evaluating the quantification and detection limit of the targeted analyte, as well as by checking the repeatability and reproducibility of our designed sensor. The sensitivity of our modified electrode (MWCNTs/Cu–ZnO/GCE) was determined by recording SWVs of different concentrations of CBB R-250, starting from a higher concentration of 30 μM to a lower concentration of 0.02 μM using the pre-optimized experimental conditions, *i.e.*, 0.3 M H_2_SO_4_, 0.3 V accumulation potential and 20 s of accumulation time. SW voltammograms were recorded for all these concentrations in a stepwise manner, as represented in Fig. 5A.

The linearity plot was established using the lower concentrations data of CBB R-250, as depicted in Fig. 5B. The limit of detection (LOD) of 0.109 nM and quantification (LOQ) of 0.369 nM were calculated using the following equations.^28^4
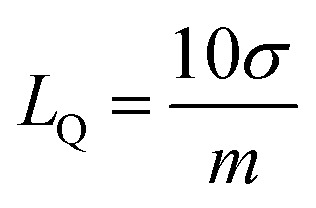
5
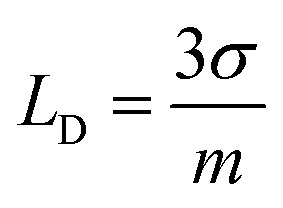
Here, *σ* symbolizes the standard deviation determined from the peak current value of the blank solution (twelve runs) and *m* indicates the slope that was obtained from the calibration plot of lower concentrations.

#### 3.4.2. Assessment of the stability of the developed sensing scaffold

The stability of the developed sensor (MWCNTs/Cu–ZnO/GCE was evaluated by checking its repeatability and reproducibility of voltammetric response under the pre-optimized conditions. In order to check the reproducibility of our designed sensor, five different electrodes having the same dimensions were modified and SWVs were performed in each case. No significant change was recorded in the peak current of CBB R-250, as indicated in Fig. S7A.† Similarly, in order to check the repeatability of our designed sensor, a single GCE was modified at different time intervals and SWVs were recorded under the pre-optimized conditions. The results revealed that no prominent change happened in the oxidation peak current of CBB R-250, as evident from Fig. S7B.† From the results obtained through reproducibility and repeatability, it can be concluded that our designed sensor is quite reliable and stable over a long period.

### 3.5. Photodegradation of CBB R-250

The following section presents a detail of the photodegradation of CBB R-250.

#### 3.5.1. Spectroscopic and voltammetric monitoring of dye degradation

**Fig. 6:**
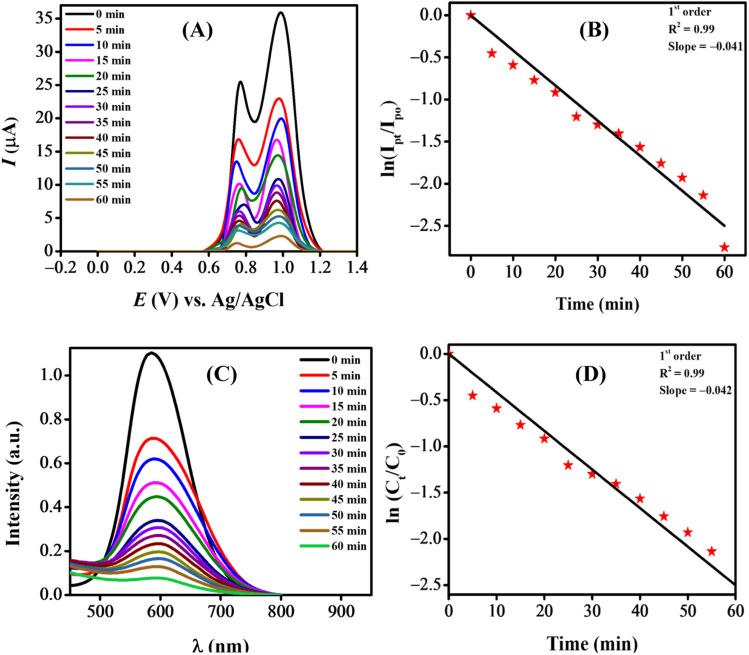
NPs-assisted photo-Fenton degradation of the dye. (A) Electrochemical monitoring; (C) UV-vis spectroscopic monitoring; kinetic studies of degradation using (B) SW voltammetric data; (D) UV-vis spectrophotometric data.

The NPs-assisted Fenton-based photodegradation of the CBB R-250 was monitored using the SWV technique. The required amount of solution was obtained from the reaction mixture every 5 min, and a drop from that solution was cast upon the modified GCE. SWVs were recorded using MWCNTs/Cu–ZnO/GCE under the pre-optimized conditions (0.3 M H_2_SO_4_, deposition potential 0.3, deposition time 20 s) until complete degradation of the dye was achieved. Fig. 6A represents the SWVs of the CBB R-250 degradation over time. Since the concentration is directly proportional to the current, a gradual decrease in the current response of CBB R-250 was recorded. This is an indication of the photocatalytic degradation of CBB R-250. The extent of degradation (% degradation) was evaluated using the following relationship.6
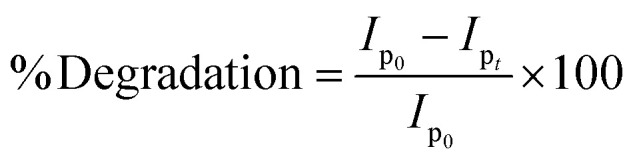
where, *I*_p_0__ represents the initial peak current of the dye, *i.e.*, peak current at time “0” min and *I*_p_*t*__ represents the peak current at time “*t*”. Fig. S8A† shows the % degradation of the CBB R-250 dye. It can be seen that the degradation is initially much faster, and slows down with the passage of time. This can be attributed to there being a lower number of active sites available at the latter half of the reaction. The maximum degradation efficiency in the present case was calculated to be 96%. Kinetic analysis of the CBB R-250 photodegradation was also carried out using SWV data. From Fig. 6B, it is evident that the data fit best into pseudo-first-order kinetics. The rate constant “*k*” for the reaction was calculated using the following straight–line relationship.^20^7
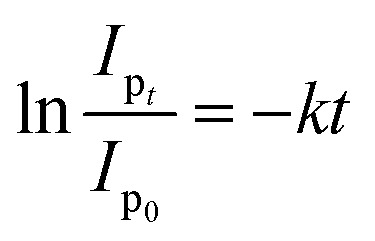
where *I*_p_0__ and *I*_p_*t*__ correspond to the peak current at time zero and time “*t*”, respectively. From the slope of the graph, the “*k*” value for the CBB R-250 degradation was calculated to be 0.041 min^−1^.

The NPs-assisted Fenton-based photodegradation of CBB R-250 was also monitored employing UV-vis spectrophotometry. The required amount of solution was obtained from the reaction mixture every 5 minutes, and UV-vis spectra were recorded until the dye completely degraded. Fig. 6C represents the absorption spectra of the CBB R-250 dye degradation over the passage of time. As the concentration is directly proportional to the absorbance, a gradual decrease in the absorption spectra of the dye was recorded, indicative of the photocatalytic degradation of the dye. The extent of degradation (% degradation) was calculated using the following relationship:^29^8
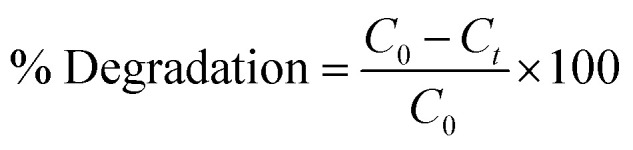
where, *C*_0_ represents the initial absorbance of the dye, *i.e.*, absorbance at time “0” min, and *C*_*t*_ represents the absorbance at time “*t*”. The percentage degradation of the CBB R-250 dye is represented in Fig. S8B.† It can be seen that the degradation is initially much faster, which slows down with the passage of time. This can be related to the lower number of active sites being available at the latter half of the reaction. The maximum degradation efficiency in the present case was calculated to be 96%.

Kinetic analysis of the CBB R-250 photodegradation was also studied using UV-vis spectroscopic data. From Fig. 6D, it is evident that the data best fit into pseudo-first-order kinetics. The rate constant “*k*” for the reaction was calculated using the following straight-line relationship.9
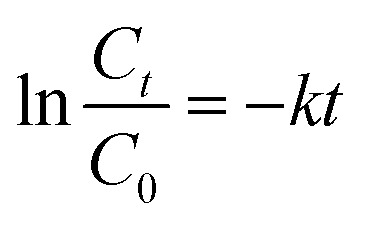
where *C*_0_ and *C*_*t*_ correspond to the absorbance at time zero and time “*t*”, respectively. From the slope of the graph, the “*k*” value for the CBB R-250 degradation was calculated to be 0.042 min^−1^.

As depicted in Fig. S9,† when the Cu–ZnO NPs were used alone in the absence of the Fenton reagent, a 65% degradation in 105 minutes with a rate constant value of 0.001 min^−1^ was obtained. Photocatalytic degradation of the dye was also monitored with the Fenton reagent alone to consider the role provided by Cu–ZnO NPs during the degradation process. Fig. S10A† demonstrates the UV-vis absorption spectra of the photo-Fenton degradation of the CBB R-250 dye. The extent of degradation as represented in Fig. S10B† was calculated to be 90%. The time taken for degradation was also more than that of the nanoparticles-assisted photo-Fenton reaction. So overall, it can be concluded that NPs play a key role in enhancing the extent of degradation, as well as minimizing the time required for complete degradation. It can be attributed to there being a greater number of active sites provided by the NPs for adsorption, so the generated free radicals can easily target their host and initiate the degradation process.

**Fig. 7:**
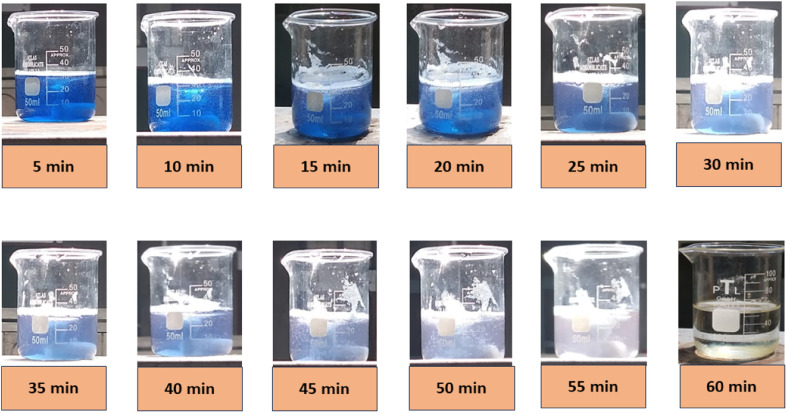
Visual evidence of CBB R-250 photodegradation by Cu–ZnO/Fe^2+/^H_2_O_2_.

A naked eye observation of the CBB R-250 dye degradation is represented in Fig. 7.

#### 3.5.2. Proposed mechanism of CBB R-250 degradation

**Scheme 2:**
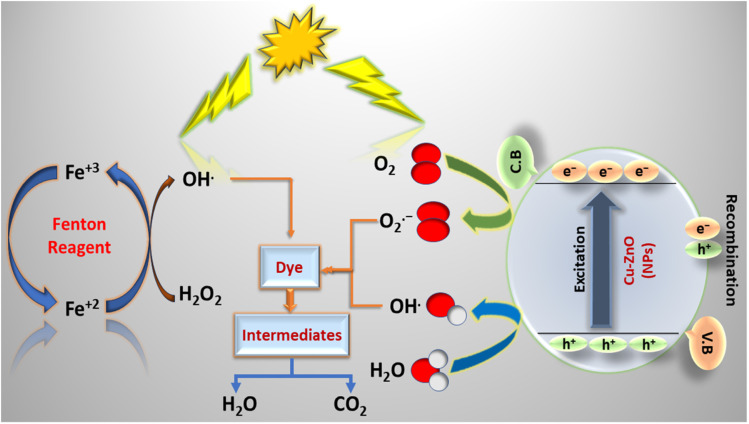
Proposed mechanism for NPs-assisted photo-Fenton degradation of the dye.

A proposed mechanism for the NPs-assisted photo-Fenton degradation of CBB R-250 is given in Scheme 2. Upon the absorption of UV-vis radiation of appropriate wavelength corresponding to the band gap of the nanoparticles, the Cu–ZnO NPs produce electron–hole pairs. Reaction with the air O_2_ and water then results in the subsequent production of O_2_˙^−^ and HO˙. The reactions have been represented in eqn (10)–(15). The reactive species generated through these reactions,particularly HO˙, participate in the photodegradation of the dye through a series of oxidation reactions.^20^

10 Cu–ZnO + *hv* → e^−^ + h^+^

11 e^−^ + O_2_ → O_2_˙^−^

12 h^+^ + H_2_O → OH˙ + H^+^

13 H^+^ + O_2_˙^−^ → HOO˙

14 HOO˙ + HOO˙ → H_2_O_2_ + O_2_

15 H_2_O_2_ → 2OH˙

Similarly, Fe^2+^ present in the Fenton reagent reduces hydrogen peroxide to form hydroxyl radical and hydroxyl ion, respectively. Fe^3+^ generated in the first step reacts with H_2_O_2_ to form hydroperoxyl radical and H^+^. These two reactions produce water as a by-product. Overall, the photo-Fenton reaction produces reactive HO˙, which actively participates in the degradation of CBB R-250. The subsequent reactions are represented by eqn (16)–(19) respectively.^30,31^

16 H_2_O_2_ + Fe^2+^ → HO˙ + HO^−^ + Fe^3+^

17 Fe^3+^ + H_2_O_2_ → Fe^2+^ + HOO˙ + H^+^

18 Fe^3+^ + HOO˙ → Fe^2+^ + O_2_ + H^+^

19 H_2_O_2_ + *hv* → 2HO˙

The reactive species generated through both of these process results in the enhanced degradation of the dye compared to their individual effects. These radicals first convert the dye into different simpler organic compounds (like alcohol, aldehyde, ketone), and finally into water and carbon dioxide. Furthermore, the nanoparticles also allow the dye to be adsorbed over its surface, which makes it more prominent for degradation through the photo-Fenton reaction.

### 3.6. Adsorption studies of the dye using ZnO NPs

Adsorption of CBB R-250 dye was achieved using ZnO NPs. Different conditions like the adsorbent concentration, volume of dye solution, pH of the solution, and contact time were optimized to get the best results. For monitoring of the CBB R-250, the UV-vis spectroscopic technique was employed. Prior to the adsorption of the dye, a calibration plot was obtained for the dye solution, having known concentrations with that of its UV-vis absorbance. The calibration plot of the dye concentration *vs.* UV-vis absorbance is presented in Fig. S11.† For the adsorption studies, UV-vis spectra were recorded at regular time intervals, and then the absorbance of dye was converted into its concentration (in ppm) using the calibration plot. The following equation was used to study the maximum dye removal efficiency of the NPs.^32^20
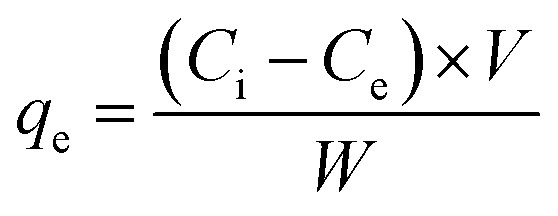
where, *q*_e_, *V*, *W*, *C*_i_, and *C*_e_ are the maximum adsorption capacity (mg g^−1^), solution volume (L), mass of NPs in grams, initial dye concentration (mg L^−1^), and equilibrium dye concentration (mg L^−1^), respectively. Different kinetic models were employed to study the mechanism of mass transfer between the liquid and solid phases. Similarly, adsorption isotherms were employed to get into the depth of the adsorption mechanism. The details of adsorption studies are given below.

#### 3.6.1. Spectroscopic monitoring of dye adsorption

**Fig. 8:**
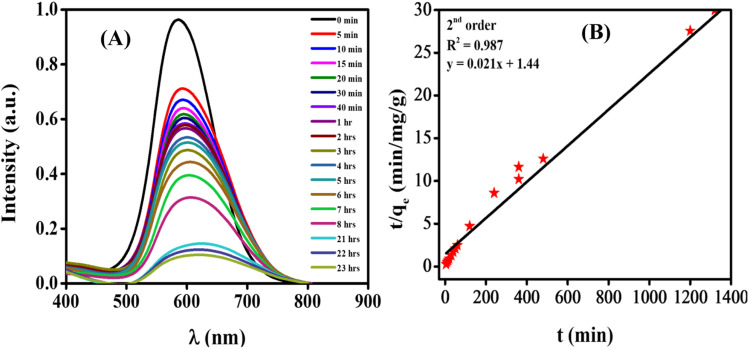
(A) UV-vis spectroscopic monitoring of dye adsorption; (B) kinetic studies of adsorption using a 2nd order equation.

For the adsorption study of CBB R-250, the required amount of ZnO NPs was introduced to the dye solution, and it was covered completely with aluminum foil to remove the possibility of any sort of light-driven photocatalytic reaction. Under the optimized conditions of adsorbent concentration (10 mg), contact time (23 hours), volume of the dye (25 mL), and pH of the solution (pH = 3), the adsorption of CBB R-250 dye with ZnO NPs was monitored using UV-vis spectroscopic technique. For that purpose, an equal volume of the dye solution was obtained from the reaction mixture after an interval of time, and UV-vis spectra were obtained in each case. The UV-vis spectroscopic monitoring of dye adsorption is shown in Fig. 8A. The maximum adsorption capacity (*q*_e_) was calculated in each case using eqn (20), and subsequent kinetic and adsorption isotherm analysis were performed.

Table 1 presents a brief comparison of the degradation and adsorption results from the current work with the literature reported data. Our catalyst was promising for the removal of CBB R-250 dye as it works efficiently in neutral medium, while the reported catalysts are effective mostly in alkaline media.

Comparison of the obtained degradation and adsorption results with reported literature

**Table:** 

Degradation				
Materials	Degradation efficiency (%)	Medium	Time (min)	References
CuO NPs	58	—	90	15
ZnO-decorated polypyrrole/chitosan	92	—	70	33
ZnO-NPs	93	—	180	4
Ag NPs	93	Alkaline	30	34
α-MnO_2_/TiO_2_	98	Alkaline	30	35
Cu–ZnO/Fe^2+^/H_2_O_2_	96	Neutral	60	Present work

**Table:** 

Adsorption		
Materials	Adsorption capacity (mg g^−1^)	References
Iron oxide-graphene oxide composites	14	16
Starch/poly(alginic acid-*cl*-acrylamide) nanohydrogel	31	17
ZnO NPs	48	Present work

#### 3.6.2. Kinetic studies

A kinetic analysis was conducted to examine the rate of the adsorption process, and assess the mechanism of mass transfer from the liquid to the solid adsorbent. Various kinetic models were utilized, namely pseudo-second-order kinetics (Fig. 8B), pseudo-first-order kinetics, and intra-particle diffusion model, as depicted in Fig. S12.† The equation used is given as,21
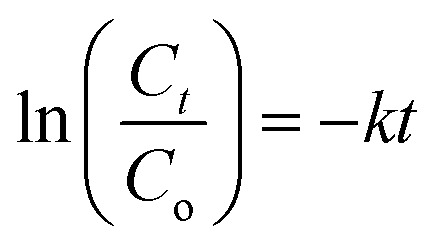
where ln(*C*_*t*_/*C*_o_) is on the *y*-axis, *t* is on the *x*-axis and *k* is the slope equal to the rate constant. *C*_o_ corresponds to the initial concentration and *C*_*t*_ corresponds to the concentration at time *t*.

The pseudo-second-order kinetics equation is given below.^32^22
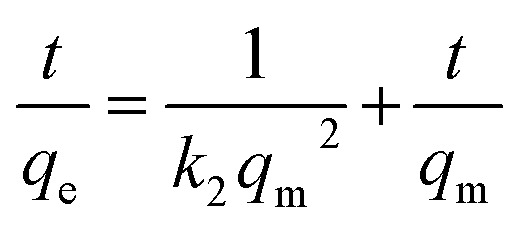
where *t*/*q*_e_ is on the *y*-axis, *t* is on the *x*-axis, 1/*q*_m_ is the slope and 1/*k*_2_*q*_m_^2^ is the intercept. *q*_m_ corresponds to the maximum adsorption capacity, *k*_2_ is the rate constant, and *q*_e_ denotes the adsorption capacity at a specific time. Similarly, the straight-line form of the intra-particle diffusion model has been given as:

23 *q*_e_ = *k*_pi_*t*^1/2^ + *C*_i_

Here, *q*_e_ is on the *y*-axis, *t*^1/2^ on the *x*-axis, *k*_pi_ (mg g^−1^ min^−1^) is the slope, and *C*_i_ is the intercept, while *q*_e_ is the adsorption capacity at a specific time. The value of the co-relation coefficients (*R*^2^) was calculated for all of these, while the highest was observed for the pseudo-second order reaction. It was concluded that adsorption of CBB R-250 follows pseudo-second order kinetics, and the rate is dependent on the square of active sites available. The value of *q*_m_ was calculated to be 47.61 mg g^−1^, while the rate constant *k*_2_ was equal to 0.00031 g mg^−1^ min^−1^.

#### 3.6.3. Equilibrium studies

The data obtained *via* UV-vis spectroscopy for the adsorption of the CBB R-250 dye were fitted into three different isotherm models to better understand the nature of the surface (homogeneity and heterogeneity) of the adsorbent, and the possible interactions between the adsorbent and adsorbate molecules. Different isotherms were applied to the given data of adsorption, namely Langmuir adsorption isotherm, Freundlich adsorption isotherm, and Temkin adsorption isotherm.^32^ The linear form of these isotherms has been given as:24
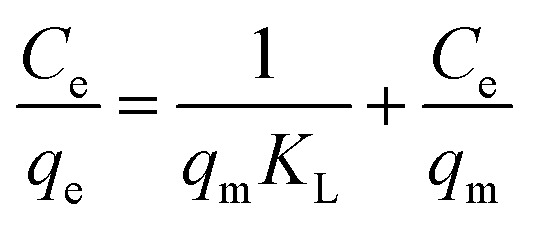
25
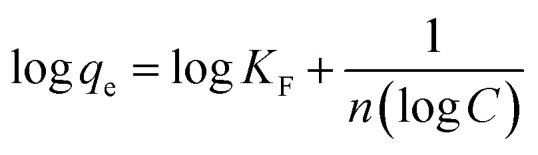

**Fig. 9:**
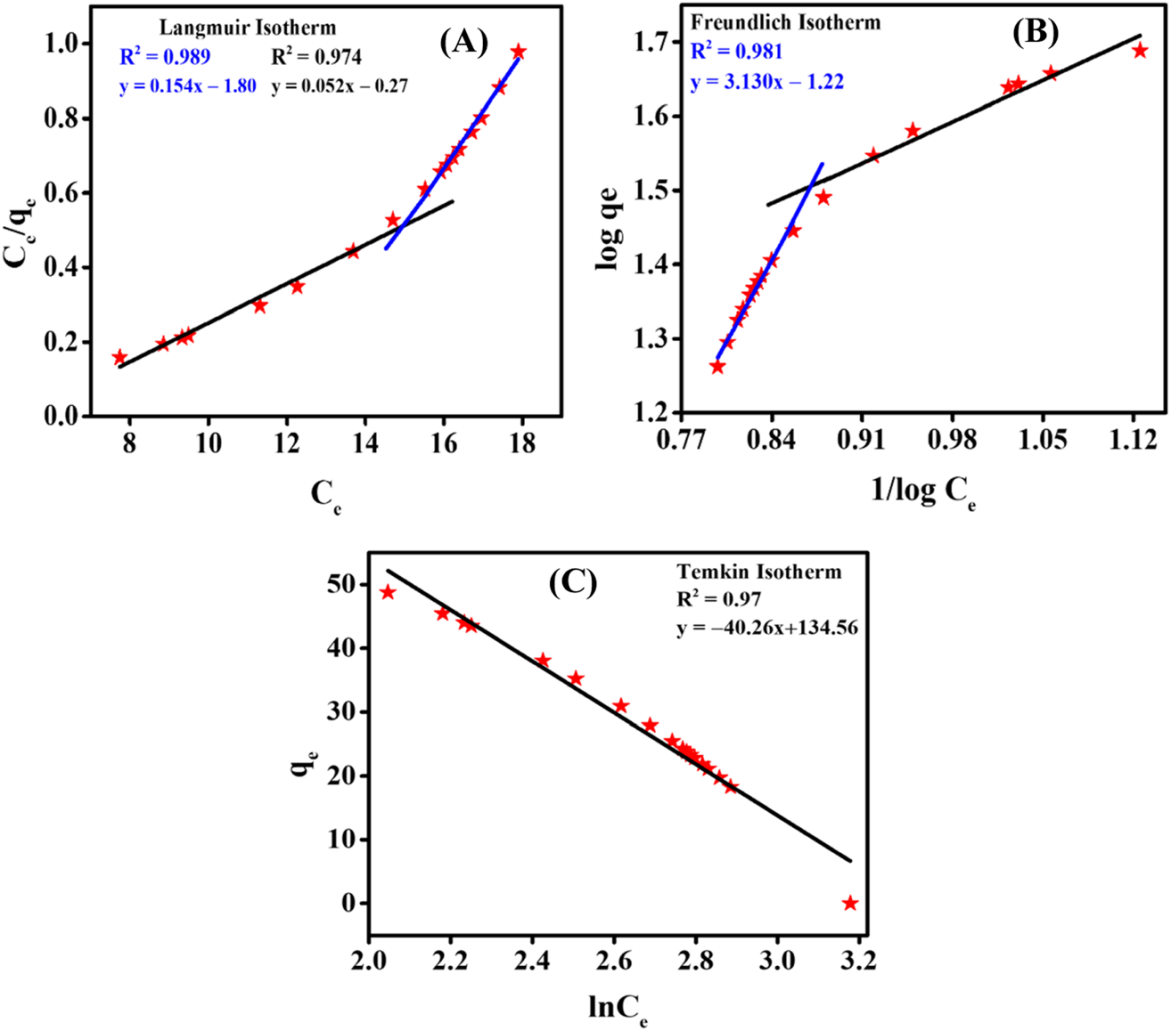
(A) Langmuir adsorption isotherm; (B) Freundlich adsorption isotherm; (C) Temkin isotherm.

26 *q*_e_ = *B* ln *A* + *B* ln *C*

where eqn (24)–(26) correspond to the Langmuir, Freundlich, and Temkin isotherms, respectively. *q*_e_ corresponds to the adsorption capacity at a specific time, *q*_m_ corresponds to the maximum adsorption capacity, *K*_L_ and *K*_F_ correspond to Langmuir and Freundlich constants, respectively, *n* is the intensity, *C*_e_ is the CBB R-250 concentration at time *t*, *C* is the concentration of adsorbate at equilibrium, *B* = *RT*/*b*, and *A* corresponds to the equilibrium binding constant. The various types of isotherms applied to the data obtained for CBB R-250 adsorption are represented in Fig. 9. The elucidation of the fundamental characteristics pertaining to the adsorption phenomena can be facilitated through the utilization of a dimensionless parameter known as the separation factor *R*_L_, which is derived from the Langmuir constant.^32^27
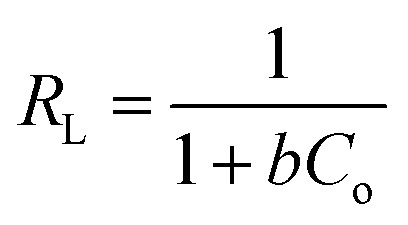
where *C*_o_ is the initial concentration of the analyte (mg L^−1^). If the value of *R*_L_ is between 0 and 1, it means that adsorption is favorable, while a value greater than 1 corresponds to unfavorable adsorption. A value equal to 0 means irreversible adsorption.

It was observed that at a higher concentration of dye, the data were best fitted into the Freundlich adsorption isotherm. At a lower concentration of adsorbate, the data were best fitted into the Langmuir adsorption isotherm. Meanwhile, it followed the Temkin isotherm over the full range of concentration. This observed unique adsorption behavior, where the CBB R-250 dye on ZnO NPs follows Freundlich isotherms at higher concentrations and switches to Langmuir isotherms at lower concentrations while consistently conforming to the Temkin isotherm across the entire range, can be attributed to the evolving surface interactions and the availability of adsorption sites. At higher dye concentrations, the Freundlich isotherms predominate, signifying multilayer adsorption due to the saturation of surface sites as more dye molecules are introduced. Conversely, at lower concentrations, the Langmuir isotherms become dominant, reflecting the monolayer adsorption on a surface less saturated with dye molecules. This transition underscores the changing nature of adsorbate–adsorbent interactions with varying concentrations, while the Temkin isotherm, which considers the heat of adsorption alterations, consistently fits the observed data, further supporting the complex interplay of surface chemistry and adsorption energetics. The respective values of parameters obtained for different isotherms are given in Table 2.

**Table:** Parameters obtained from different adsorption isotherms

Isotherm	Parameter	Value
Langmuir	*q*_m_	19.23 mg g^−1^
	*K*_L_	0.192 L mol^−1^
	*R*_L_	0.178 unitless
Freundlich	*N*	0.319 unitless
	*K*_F_	16.59 mg g^−1^
Temkin	*B*	18.42 J mol^−1^
	*A*	28.28 L g^−1^

## 4. Conclusions

ZnO and Cu–ZnO NPs were successfully phytosynthesized using a green synthesis approach. The addition of copper as a dopant led to improvement in the photocatalytic, electrochemical, and optical behavior of the synthesized Cu–ZnO NPs. The as-synthesized NPs along with MWCNTs were employed to modify the GCE to enhance its detection performance for CBB R-250. The voltammetric performance of MWCNTs/Cu–ZnO/GCE towards the sensing of CBB R-250 was further boosted through the optimization of the supporting electrolyte, electrolyte concentration, deposition time, and deposition potential. The designed sensor helped in detecting CBB R-250 up to 0.1 nM concentration under optimized conditions. The findings of cyclic voltammetry and impedance spectroscopy revealed that the synergistic combination of Cu–ZnO and MWCNTs enhance the electroactive surface area and minimize the charge transfer resistance as required for efficient electron transport through the designed sensing platform. The stability was ensured by the reproducibility and repeatability tests. The synthesized NPs were also employed for the photodegradation of CBB R-250. The synergistic effect of Cu–ZnO NPs together with the Fenton reagent demonstrated 96% removal of CBB R-250 dye in neutral medium in 1 hour. The photodegradation of CBB R-250 followed pseudo-first-order kinetics. The results of the degradation kinetics evaluated *via* SWV and UV-vis spectroscopy were found to be in good agreement. ZnO NPs were employed for the adsorptive removal of CBB R-250 by optimizing different conditions of adsorbent dose, pH, and concentration of dye. It was found that the synthesized NPs demonstrated the maximum adsorption capacity of 48 mg g^−1^ under optimized conditions. Adsorption of CBB R-250 followed the Langmuir isotherm at lower concentration and the Freundlich isotherm at higher concentration.

## Conflicts of interest

The authors declare no competing financial or non-financial interests.

## Supplementary Material

RA-014-D3RA07519B-s001

## References

[cit1] Sriram G., Bendre A., Mariappan E., Altalhi T., Kigga M., Ching Y. C., Jung H.-Y., Bhaduri B., Kurkuri M. (2022). Sustainable Mater. Technol..

[cit2] Khan K. A., Shah A., Nisar J., Haleem A., Shah I. (2023). Molecules.

[cit3] Priya B. S. K., Gupta U. S. B., Bhatia J. K. (2018). React. Funct. Polym..

[cit4] Singh K., Singh J., Rawat M. (2019). SN Appl. Sci..

[cit5] Shad N. A., Zahoor M., Bano K., Bajwa S. Z., Amin N., Ihsan A., Soomro R. A., Ali A., Imran Arshad M., Wu A., Iqbal M. Z., Khan W. S. (2017). Inorg. Chem. Commun..

[cit6] Ezzat A. O., Tawfeek A. M., Rajabathar J. R., Al-Lohedan H. A. (2022). Molecules.

[cit7] Irfan M., Shah A., Iftikhar F. J., Hayat M., Ashiq M. N., Shah I. (2022). ACS Omega.

[cit8] Xu P. S., Sun Y. M., Shi C. S., Xu F. Q., Pan H. B. (2003). Nucl. Instrum. Methods Phys. Res., Sect. B.

[cit9] Kou L. Z. , Guo W. L. and Li C., 2008 Symp. Piezoelectricity, Acoust. Waves, Device Appl. SPAWDA 2008, 2008, pp. 354–359

[cit10] Zhang Z., Yi J. B., Ding J., Wong L. M., Seng H. L., Wang S. J., Tao J. G., Li G. P., Xing G. Z., Sum T. C., Huan C. H. A., Wu T. (2008). J. Phys. Chem. C.

[cit11] Peng X., Xu J., Zang H., Wang B., Wang Z. (2008). J. Lumin..

[cit12] Ajmal A., Majeed I., Malik R. N., Idriss H., Nadeem M. A. (2014). RSC Adv..

[cit13] Saratale R. G., Saratale G. D., Chang J. S., Govindwar S. P. (2011). J. Taiwan Inst. Chem. Eng..

[cit14] Jaafarzadeh N., Takdastan A., Jorfi S., Ghanbari F., Ahmadi M., Barzegar G. (2018). J. Mol. Liq..

[cit15] Venkata A. L. K., Anthony S. P., Muthuraman M. S. (2019). Mater. Res. Express..

[cit16] Kaur N., Singh J., Kaur G., Kumar S., Kukkar D., Rawat M. (2019). Micro Nano Lett..

[cit17] Sharma G., Naushad M., Kumar A., Rana S., Sharma S., Bhatnagar A., Stadler F. J., Ghfar A. A., Khan M. R. (2017). Process Saf. Environ. Prot..

[cit18] Nayak S., Rao C. V., Mutalik S. (2022). Chem. Pap..

[cit19] Talam S., Karumuri S. R., Gunnam N. (2012). ISRN Nanotechnol..

[cit20] Sadiq M. U., Shah A., Nisar J., Shah I. (2023). Nanomaterials.

[cit21] Babu K. S. (2018). AIP Conf. Proc..

[cit22] Karthik K. V., Raghu A. V., Reddy K. R., Ravishankar R., Sangeeta M., Shetti N. P., Reddy C. V. (2022). Chemosphere.

[cit23] Jurablu S., Farahmandjou M., Firoozabadi T. P. (2015). J. Sci., Islamic Repub. Iran.

[cit24] Venu Gopal V. R., Kamila S. (2017). Appl. Nanosci..

[cit25] Bhalodia N. R., Nariya P. B., Shukla V. J. (2011). Int. J. PharmTech Res..

[cit26] Saleem M. N., Shah A., Ullah N., Nisar J., Iftikhar F. J. (2023). Catalysts.

[cit27] Mashkoor F., Nasar A., Inamuddin (2020). Environ. Chem. Lett..

[cit28] Yahya R., Shah A., Kokab T., Ullah N., Hakeem M. K., Hayat M., Haleem A., Shah I. (2022). ACS Omega.

[cit29] Aslam F., Shah A., Ullah N., Munir S. (2023). ACS Appl. Nano Mater..

[cit30] Hassaan M. A., Elkatory M. R., Ali R. M., El Nemr A. (2020). Egypt. J. Chem..

[cit31] Teng Yan Z. J., Zhang X., Liu H. (2022). Chin. J. Struct. Chem..

[cit32] Azizian S., Eris S. (2021). Interface Sci. Technol..

[cit33] Ahmad N., Sultana S., Faisal S. M., Ahmed A., Sabir S., Khan M. Z. (2019). RSC Adv..

[cit34] Venkatasubbaiah R., Jha P. K., Sanjay K. R. (2022). Chem. Eng. Commun..

[cit35] Ullah A., Rahman L., Hussain S. Z., Abbas W., Tawab A., Jilani A., Bajwa S. Z., Khan W. S., Riaz R., Hussain I., Rehman A. (2021). Mater. Sci. Eng., B.

