# Supplementary material for: Electrochemical detection and removal of brilliant blue dye via photocatalytic degradation and adsorption using phyto-synthesized nanoparticles
Source: RSC Adv. 2024 Jan 12;14(4):2504–17. doi: 10.1039/d3ra07519b (PMC10786263; doi:10.1039/d3ra07519b)
Supplement: RA-014-D3RA07519B-s001 [file RA-014-D3RA07519B-s001.pdf]

Electronic Supplementary Material (ESI) for RSC Advances.
This journal is © The Royal Society of Chemistry 2023

Electrochemical Detection and Removal of Brilliant Blue Dye via Photocatalytic Degradation and Adsorption Using Phyto-synthesized Nanoparticles

Kashif Ali Khan[a], Afzal Shah[a]*, Jan Nisar[b]

[a]Department of Chemistry Quaid-i-Azam University, Islamabad 45320, Pakistan

[b]National Centre of Excellence in Physical Chemistry, University of Peshawar, Peshawar 25120, Pakistan

Correspondence: afzals_qau@yahoo.com

Table S1: List of chemicals along with their percentage purity and supplier.

S. No.	Chemicals/Reagents	Percentage purity (mass%)	Supplier
1.	Acetic acid	99.8	Sigma-Aldrich
2.	Acetone	99	Riedel-deHaen
3.	Boric acid	>99	Sigma-Aldrich
4.	Coomassie Brilliant blue	Extra pure	Bio-Rad
5.	Copper nitrate trihydrate	>99	BDH
6.	Dimethylformamide	99	Riedel-deHaen
7.	Distilled water	--	--
8.	Disodium hydrogen phosphate dihydrate	>99	Merck
9.	Hydrochloric acid	37	Riedel-deHaen
10.	Hydrogen peroxide	35	Merck
11.	Iron nitrate hexahydrate	99	Sigma-Aldrich
12.	Iron chloride tetrahydrate	99	Merck
13.	Phosphoric acid	85	DaeJung

14.	Potassium chloride	99	Sigma-Aldrich
15.	Potassium hexacyanoferrate	>99	Sigma-Aldrich
16.	Multi-walled carbon nanotubes	--	Sigma-Aldrich
17.	Potassium hydroxide	Extra pure	Sigma-Aldrich
18.	Potassium nitrate	99	DaeJung
19.	Sodium chloride	99	Merck
20.	Sodium dihydrogen phosphate monohydrate	>99	Merck
21.	Sodium hydroxide	97	Fluka
22.	Sulphuric acid	98	BDH
23.	Zinc nitrate hexahydrate	98	BDH Analer

Table S2: Elemental composition of ZnO and Cu-ZnO NPs determined using EDS analysis.

**Materials**	**Elemental composition (wt%)**			
	**Zn**	**O**	**Cu**	**C**
ZnO NPs	76.42	19.83	--	3.75
Cu-ZnO NPs	70.06	20.38	5.42	4.14

Table S3: Calculated surface area and peak separation of the modified GCEs.

**Working electrode**	**Surface area (cm^2)**	**Peak separation (ΔE_p) (mV)**
Bare GCE	0.022	203
Cu-ZnO/GCE	0.041	174
MWCNTs/GCE	0.059	116
MWCNTs/Cu-ZnO/GCE	0.094	86

Table S4: EIS parameters obtained using model-fitted data.

Working electrode	R_s (Ω)	R_{ct} (Ω)	CPE (μF)
Bare GCE	117.2	8820	1.79
Cu-ZnO/GCE	115.6	5150	3.70
MWCNTs/GCE	115.3	1500	4.66
MWCNTs/Cu-ZnO/GCE	114.4	924.2	5.09

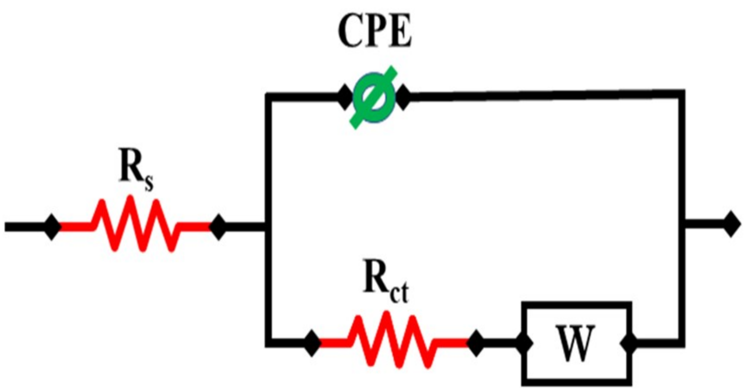

Figure S1: Equivalent circuit used for assessment of EIS parameters.

S1. Effect of scan rates

CV is a promising voltammetric method for determining the nature of voltammetric reactions that comprise either diffusion or adsorption processes. To discern the nature of reaction, it's necessary to collect CVs at different scan rates. By plotting the log of peak current against the log of scan rate, the reaction nature can be identified. From the literature, it's well established that if the slope of the plot is 0.5 or greater, diffusion likely governs the process, while a slope equal to or greater than one suggests an adsorption-controlled process.[1] The impact of varying scan rates on the electrooxidation of CBB R-250 was investigated in 0.3 M H_2SO_4 using MWCNTs/Cu-ZnO/GCE. CV responses for CBB R-250 oxidation were recorded at different scan rates ranging from 25 mVs^{-1} to 125 mVs^{-1} (Figure S2A). It can be seen that an increase in

the scan rate leads to a progressive increase in the peak current during the CBB R-250 oxidation. Meanwhile, at higher scan rates the peak potential displays a slight shift towards more positive values. This shift in peak position indicates the irreversibility of the CBB R-250 process. Similarly, the reverse scan demonstrates the absence of a cathodic peak in the CBB R-250 reduction at a lower scan rate which is also a manifestation of irreversibility of reaction. The presence of a slight peak in the reverse cycle at a higher scan rate might hint towards the presence of a short-lived intermediate which is reduced back before its further oxidation. Since the slope value for the plot between log I_p vs. log ν equals 0.80 (Figure S2B), it shows that oxidation of CBB R-250 at the surface of modified GCE has been controlled by both adsorption as well as diffusion.[2] Furthermore, since the value of regression coefficient (R^2) for the plot between I_p vs. $\upsilon^{1/2}$ (Figure S2C) is higher than that of R^2 for the plot between I_p vs. $\upsilon^{1/2}$ (Figure S2D), it suggests that oxidation of CBB R-250 governs through adsorption-controlled process. Therefore, diffusion-controlled process is less pronounced as compared to adsorption-controlled process.

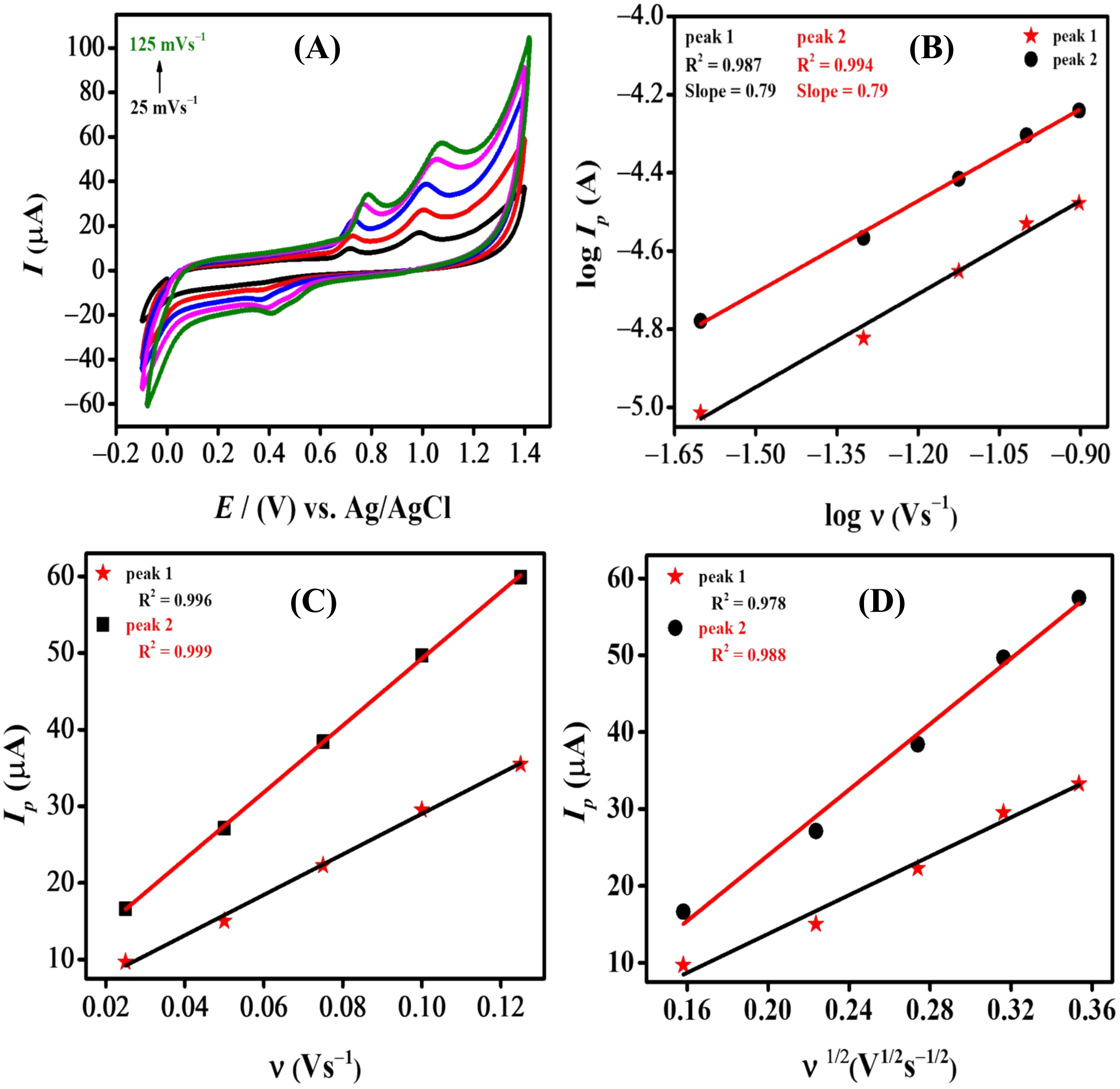

Figure S2: **(A)** Effect of various scan rates on the anodic peak current of targeted CBB R-250 in supporting electrolyte of 0.3 M H_2SO_4; **(B)** Calibration plot of CBB R-250 between log I_p vs. log ν; **(C)** Plot of peak current vs. scan rates of CBB R-250; **(D)** Calibration plot of CBB R-250 between peak current vs. square root of scan rates.

S2. Optimization of experimental conditions

There are different factors/parameters that control the peak shape, intensity, and potential of the analyte during an electrochemical experiment. In order to increase the sensitivity and selectivity of our sensing scaffold, different experimental conditions were optimized to attain the optimal value of peak current.

The stripping electrolyte substantially influences the voltammetric response of the targeted dye using modified GCE. There happens a change in ionic strength, ohmic drop, and migration current value with each of the changing electrolytes. Owing to these factors, the potential, intensity, and shape of the peak are strongly dependent on the supporting electrolyte. The oxidation current response CBB R-250 using MWCNTs/Cu-ZnO/GCE as a sensing scaffold was analyzed in different supporting electrolytes such as 0.1 M H_2SO_4, 0.1 M HCl, phosphate buffer (PBS pH=6), Britton-Robinson buffer (BRB pH=6) and 0.1 M NaCl. The effect of changing electrolytes on the peak shape and peak current of CBB R-250 is depicted in Figure S3A. As the results reveal that both the peak shape and peak current were affected by the changing electrolyte and the best current response was recorded for 0.1 M H_2SO_4. H_2SO_4 is known for its high ionic conductivity, minimal interference with the electrode material, and high stability over a broad potential range.[3] Therefore, it was chosen as the best electrolyte for the further electrochemical analysis of CBB R-250. Figure S3B represents a bar graph of the peak current (I_p) values of CBB R-250 in various supporting electrolytes.

The concentration of the electrolyte is also a determining factor in the overall sensitivity of the designed sensor toward the targeted analyte. The effect of changing the concentration of H_2SO_4 on the peak current of the targeted analyte is represented in Figure S4A. The concentration of H_2SO_4 was changed sequentially from 0.1 M to 0.6 M while its effect on the peak current of CBB R-250 was monitored. It was observed that when the concentration of H_2SO_4 increased, the peak current value increased initially until a maximum current was attained at 0.3 M, after which the peak current value decreased with further rise. This concentration corresponds to the balance between ionic conductivity, pH stability, and sensitivity. Beyond this concentration, ion over-crowding results in higher background current which results in a decline in the peak current value of the analyte.[4] This choice ensures efficient charge transfer, a consistent electrochemical environment, and maximized current signal response for accurate and reproducible CBB R-250 detection. Figure S4B represents a plot of I_p of CBB R-250 plotted against the varying concentrations of supporting electrolyte.

Deposition potential plays a vital role in determining the sensitivity and selectivity of the modified electrode and displays the overall ability of the system to quantify the targeted analyte. It determines how effectively the analyte molecules get adsorbed to the electrode surface. The

bulkier nature of most of the dyes offers steric hindrance at the surface of the electrode for the dye to be adsorbed more effectively with proper orientation. Orientation of the analyte is a decisive factor in determining its electrochemical behavior because the maximum number of analyte molecules dispose of their electroactive groups to the sensing scaffold during the redox process. By optimizing deposition potential, the sensor can then capture analyte more efficiently and effectively leading to accurate and reliable results. The deposition potential was systematically switched from −0.1 V to 0.4 V to attain the optimize current response for the specific analyte of interest (Figure S5A). An observation was made that as the deposition potential was increased towards a higher positive value, there was a corresponding increment in the peak current of CBB R-250 until it reached its maximum at 0.3 V. Beyond this value, there was a decrease in the peak current that can be attributed to the occupation of active sites at the MWCNTs/Cu-ZnO/GCE. Hence, a deposition potential of 0.3 V was selected for the subsequent electrochemical examination of CBB R-250 dye. Furthermore, the optimum voltammetric response at 0.3 V can be attributed to the anionic nature of the dye that allows the dye to interact more strongly and effectively with the sensing scaffold.[2] A plot of I_p and deposition potential is depicted in Figure S5B. Deposition time is another decisive factor in determining the sensitivity of the sensing scaffold toward the targeted analyte. The examination of the influence of deposition duration on the electrochemical oxidation of CBB R-250 involved altering the deposition time within the range of 5 seconds to 25 seconds while maintaining a constant deposition potential of 0.3 V. The graphical representation of the variation of peak current of CBB R-250 by altering the deposition time is presented in Figure S6A. Notably, the analysis revealed a progressive augmentation in analyte peak current with prolonged deposition time, peaking at 20 seconds, after which a subsequent decline in the peak current magnitude was observed. This can be related to a maximum number of active sites being occupied at this value of deposition time. So, 20 s was opted as the optimized time for further electrochemical analysis of the targeted dye as it allows for efficient and effective binding of molecules to the sensor surface. Figure S6B represents a plot of I_p of CBB R-250 vs. changing deposition time. Optimization of deposition time at 20 s shows an equilibrium between the two processes. On one hand, an increase in the deposition time allows more and more of the dye to be adsorbed or deposited to the sensor surface potentially leading to improved sensitivity. On the other hand, excessively prolonged deposition time may result in overcrowding at the surface of

MWCNTs/Cu-ZnO/GCE that might result in non-specific binding consequently hindering the interaction between analyte and sensor's active sites.[5] Therefore, 20 s of deposition time allows a maximum number of the dye molecules to be adsorbed with the best possible orientation maximizing the sensor's response to the dye.

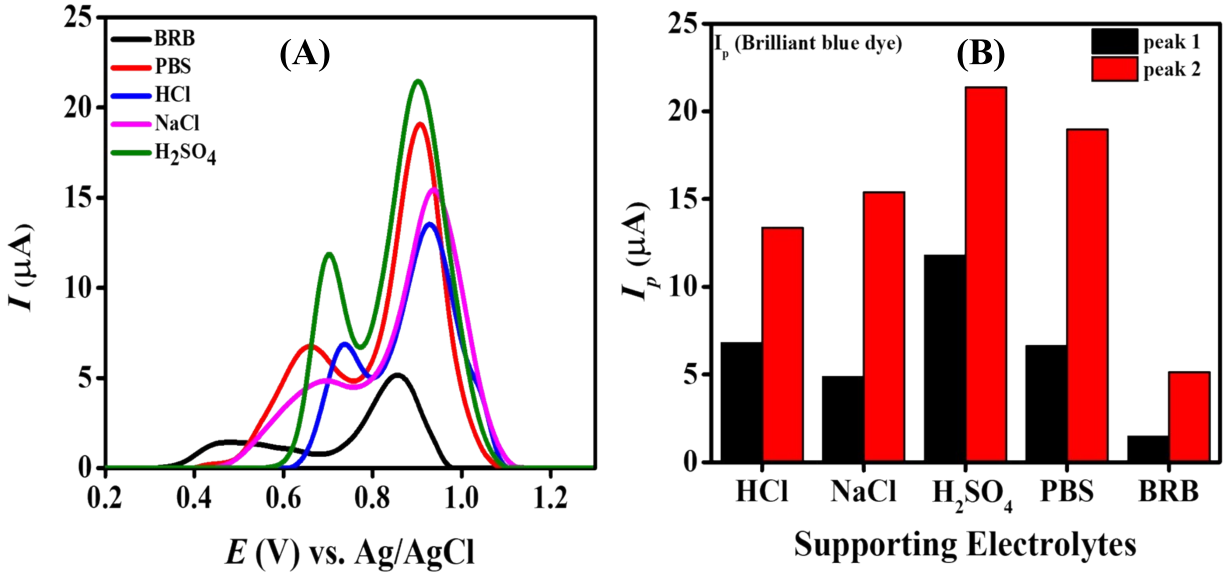

Figure S3: (A) Effect of various supporting electrolytes on the anodic peak current of CBB R-250 using MWCNTs/Cu-ZnO modified GCE; **(B)** Bar graph of CBB R-250 between peak current vs. various supporting electrolytes.

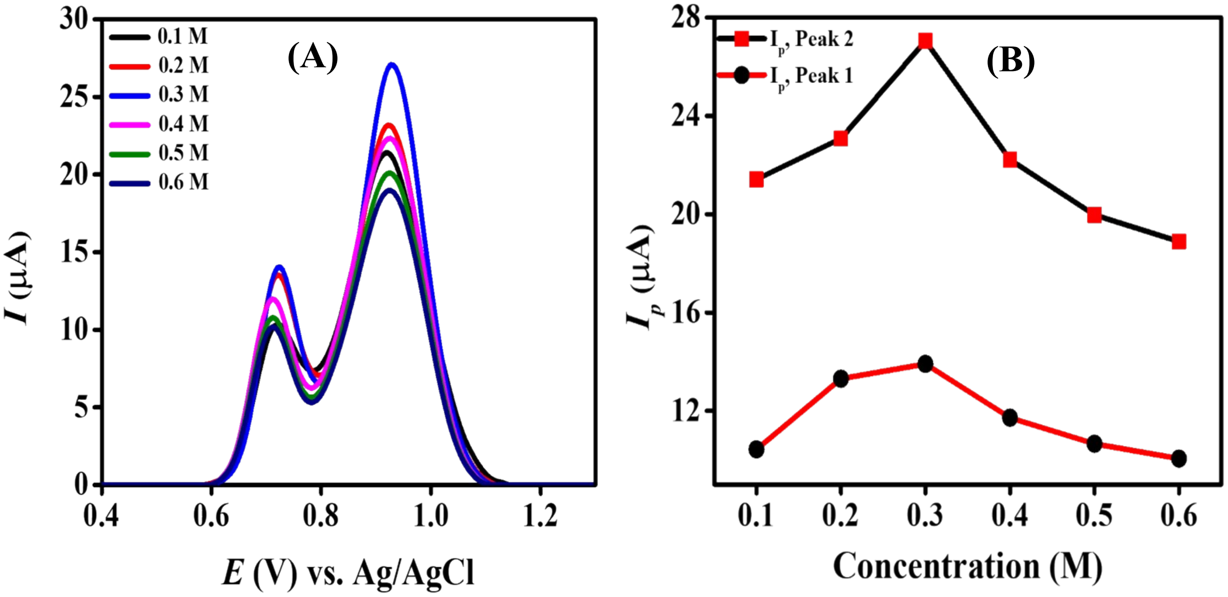

Figure S4: (A) SWVs of CBB R-250 in the solution of H_2SO_4 concentration (0.1 M-0.6 M) by using MWCNTs/Cu-ZnO as a modifier at a scan rate of 100 mVs $^{-1}$; **(B)** A plot of I_p of CBB R-250 vs. various concentrations of H_2SO_4 as supporting electrolyte.

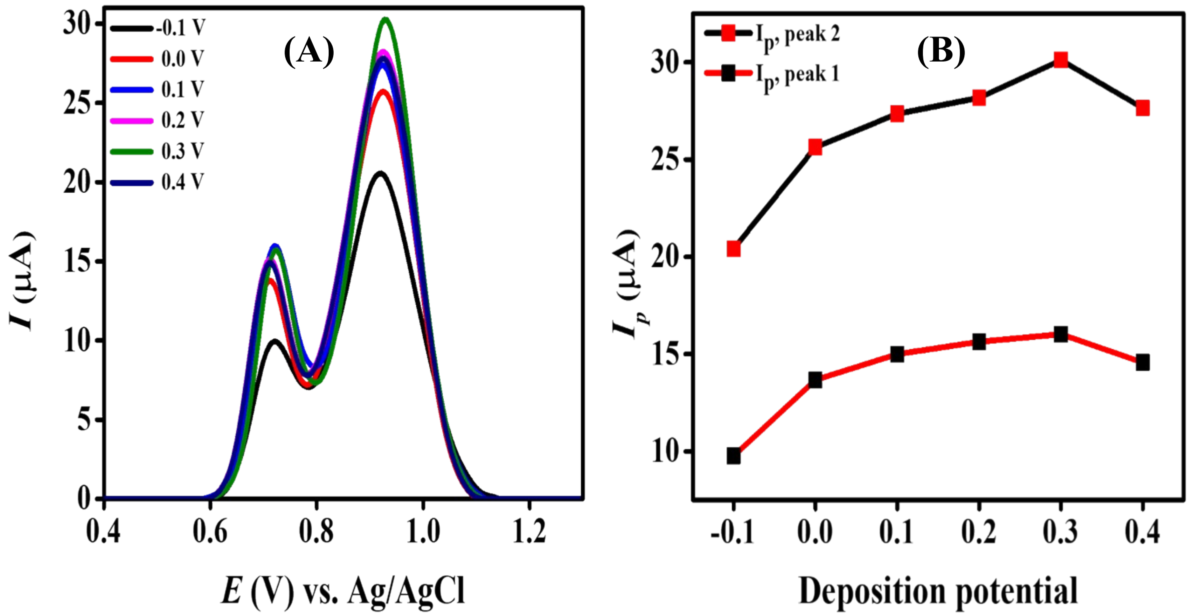

Figure S5: (A) Effect of deposition potential on the peak current of 30 µM CBB R-250 in 0.3 M H_2SO_4 using MWCNTs/Cu-ZnO/GCE; **(B)** A plot of I_p of CBB R-250 vs. deposition potential.

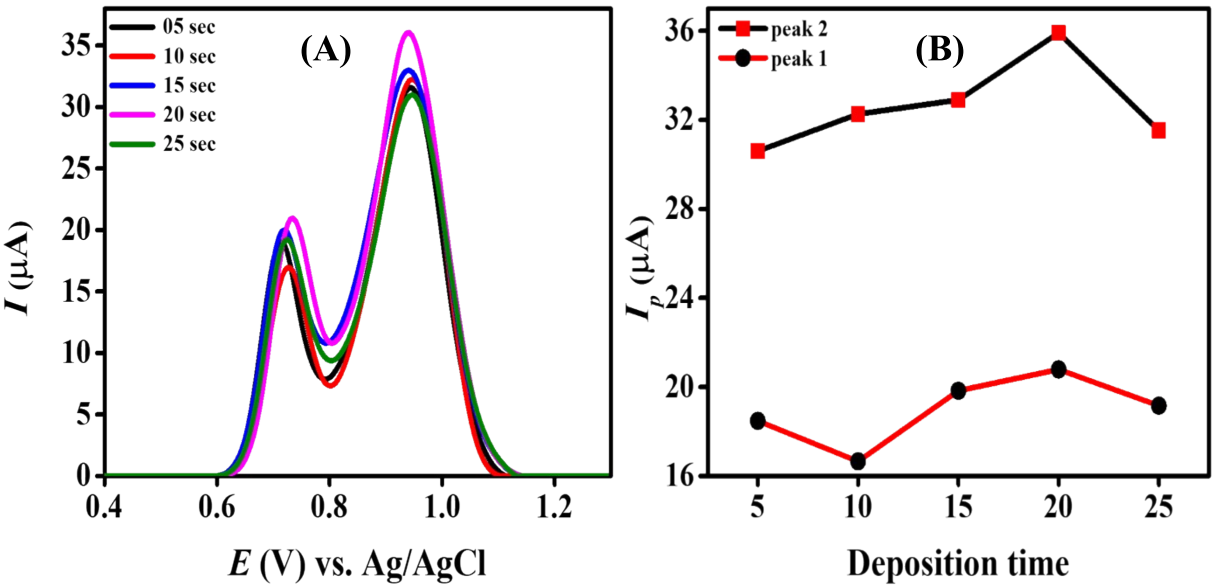

Figure S6: (A) Effect of deposition time on the peak current of 30 µM CBB R-250 in 0.3 M H_2SO_4 at a deposition potential of 0.3 V using MWCNTs/Cu-ZnO/GCE; **(B)** A plot of I_p of CBB R-250 vs. deposition time.

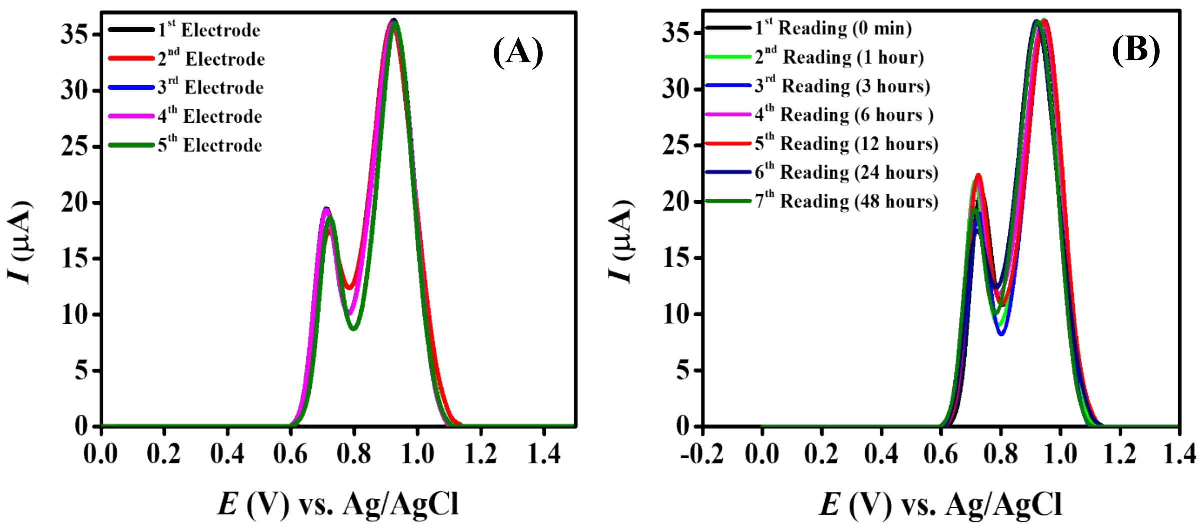

Figure S7: (A) SWVs of CBB R-250 using different modified GCEs in 0.3 M H_2SO_4; **(B)** SWVs of CBB R-250 using modified GCE at different time intervals showing repeatability of the designed sensor.

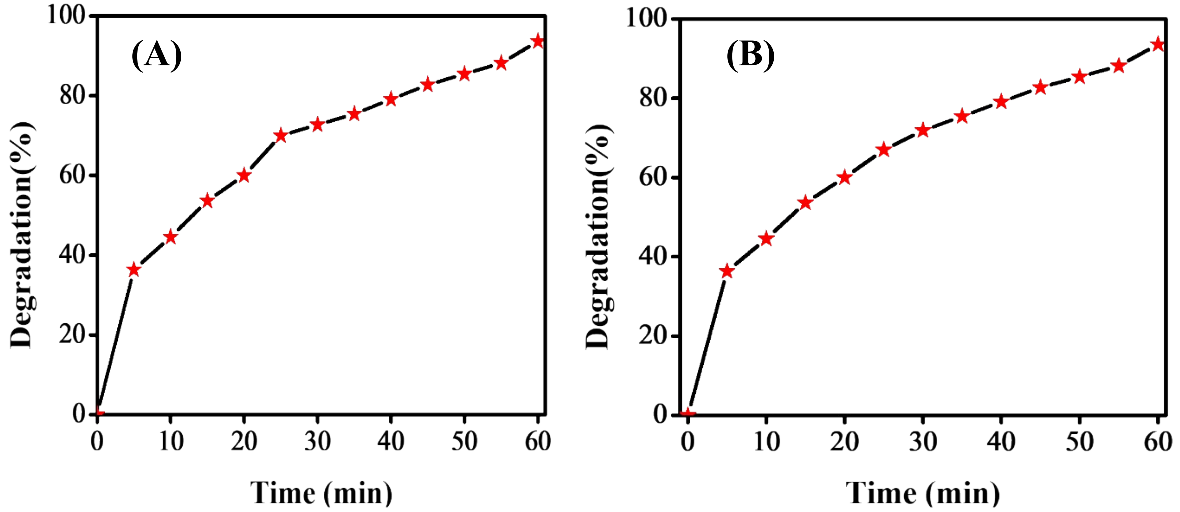

Figure S8: Plot showing extent of degradation **(A)** From SW voltammetric data; **(B)** From UV-Vis data.

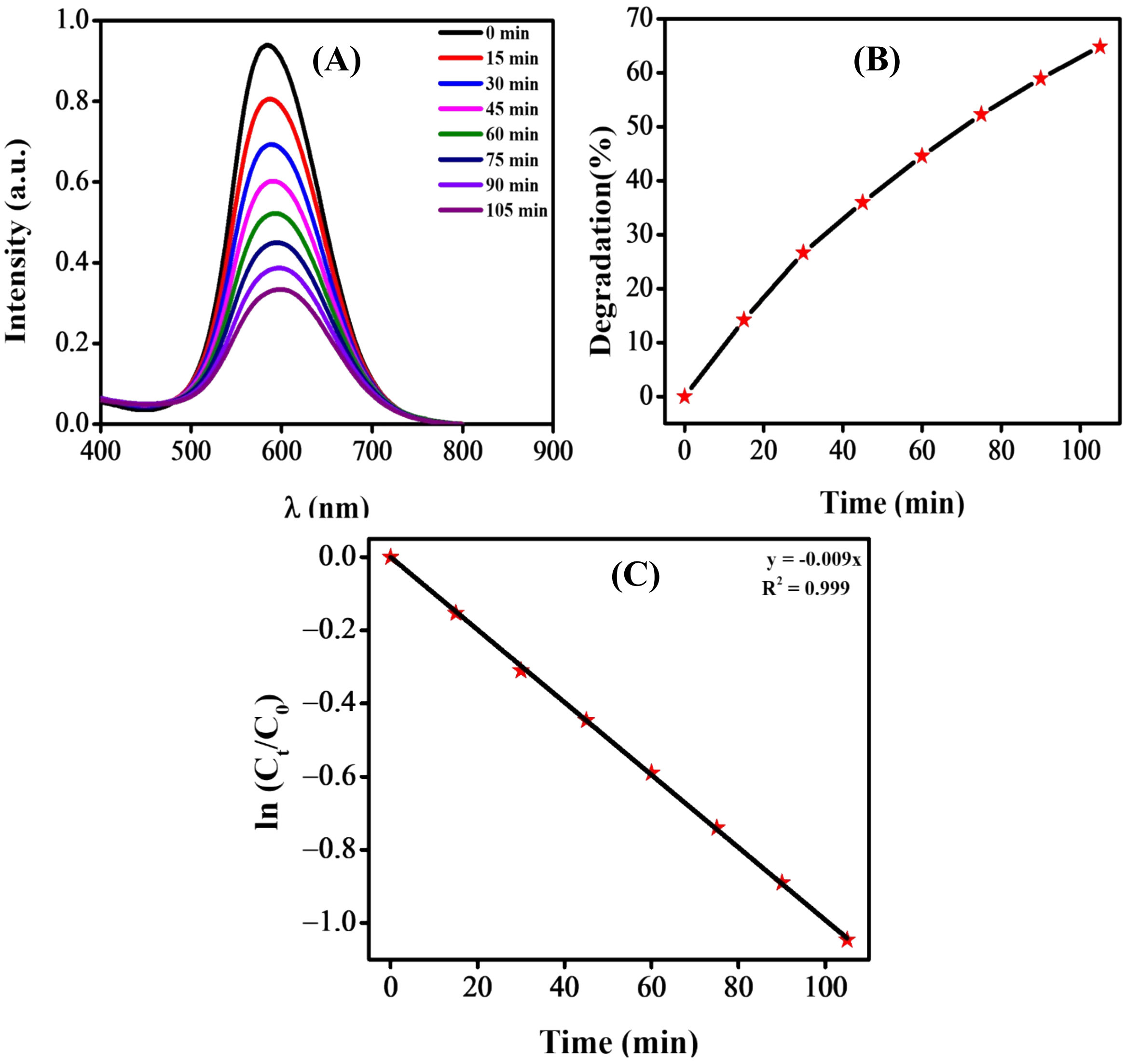

Figure S9: (A) UV-Vis. Spectroscopic monitoring of CBB R-250 dye degradation using Cu-ZnO NPs at different time intervals; **(B)** Plot showing the extent of degradation; **(C)** Kinetic studies of the degradation of CBB R-250 dye using UV-Vis. Spectroscopic data.

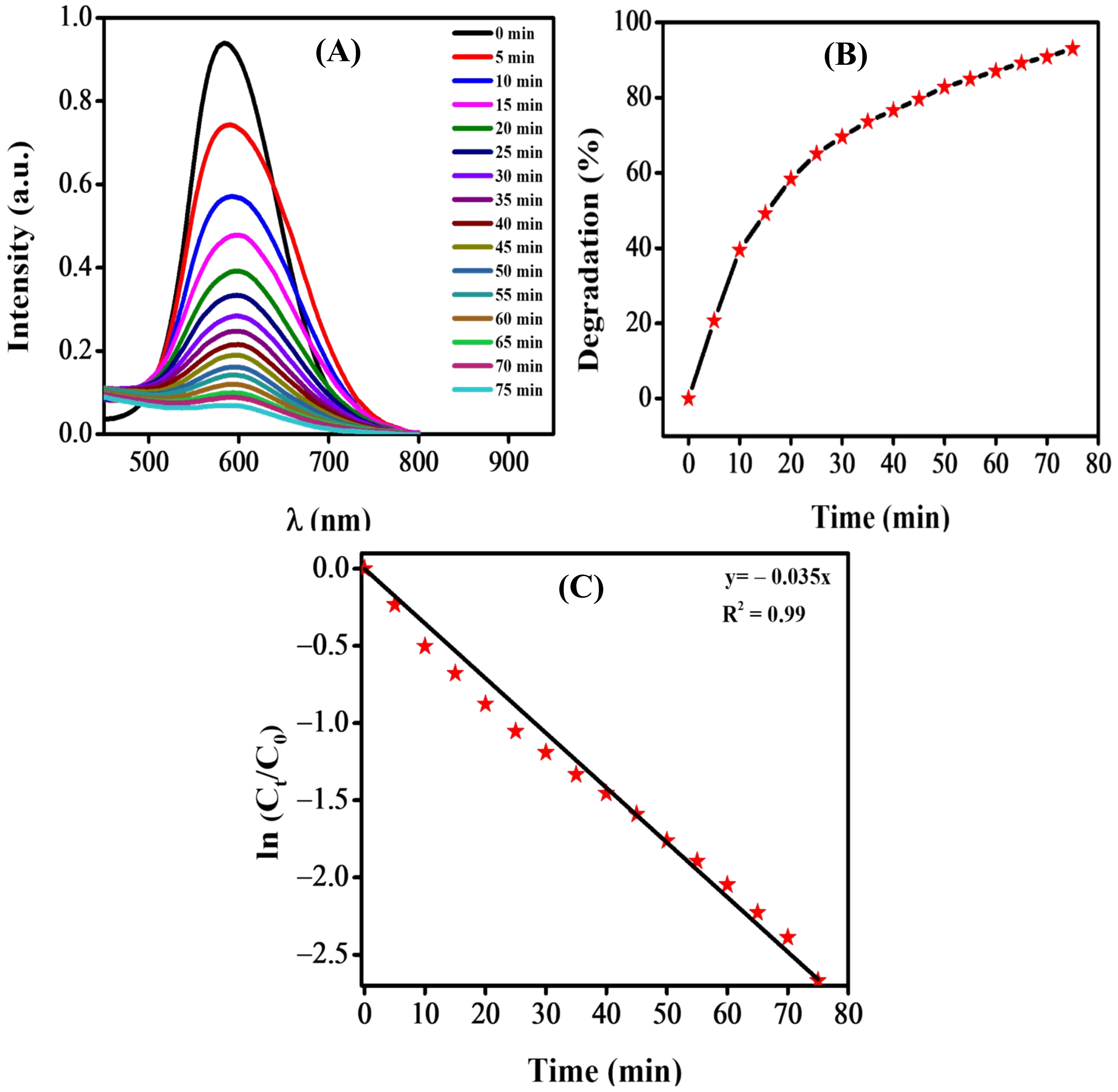

Figure S10: (A) UV-Vis spectroscopic analysis of the photo-Fenton degradation of CBB R-250; **(B)** Plot showing the extent of degradation; **(C)** Kinetic studies of the degradation of CBB R-250 dye using UV-Vis. Spectroscopic data.

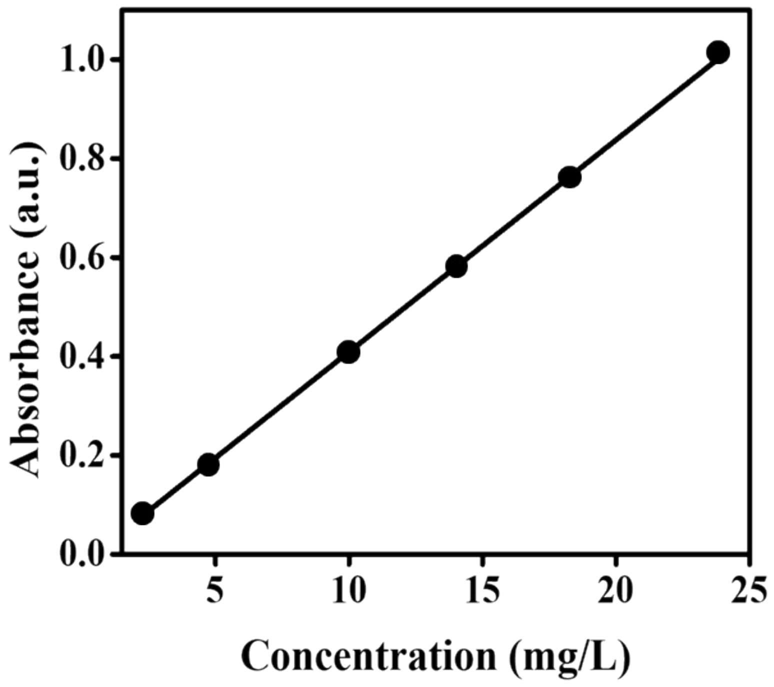

Figure S11: Calibration plot of concentration vs. absorbance.

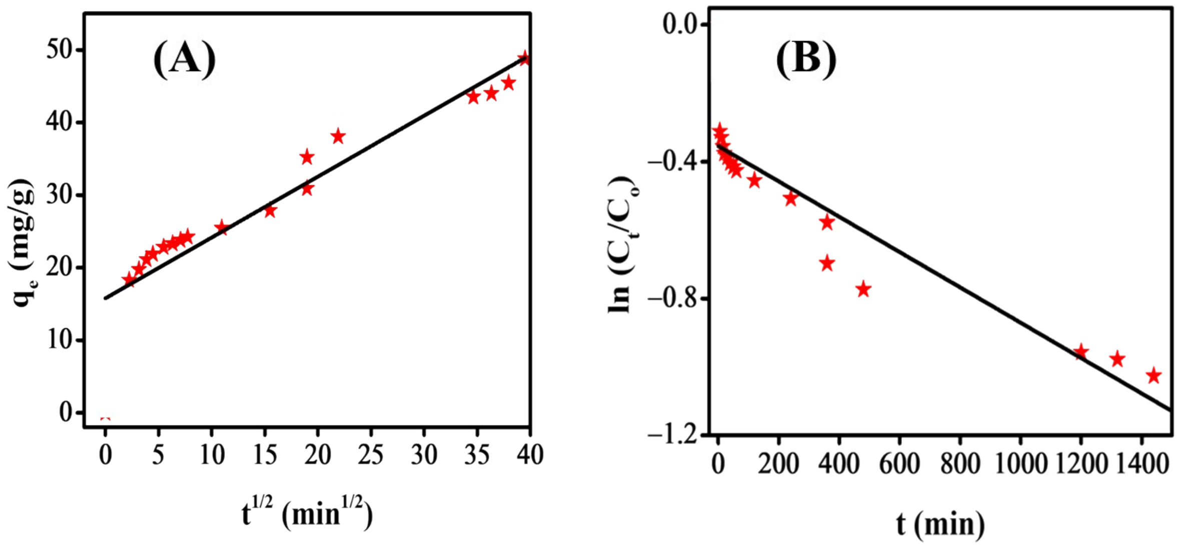

Figure S12: Kinetic studies of adsorption using UV-Vis spectroscopic data **(A**) Intra-particle diffusion model **(B)** 1st order kinetics model.

References

1 M. U. Sadiq, A. Shah, J. Nisar and I. Shah, *Nanomaterials*, 2023, **13**, 2218.

2 M. Irfan, A. Shah, F. J. Iftikhar, M. Hayat, M. N. Ashiq and I. Shah, *ACS Omega*, 2022, **7**, 32302–32312.

3 J. D. Benck, B. A. Pinaud, Y. Gorlin and T. F. Jaramillo, *PLoS One*, 2014, **9**, e107942.

4 H. Razmi, E. Habibi and H. Heidari, *Electrochim. Acta*, 2008, **53**, 8178–8185.

5 M. N. Saleem, A. Shah, N. Ullah, J. Nisar and F. J. Iftikhar, *Catalysts*, 2023, **13**, 1–18.
